# Release of an encrypted, highly potent ACE-inhibitory peptide by enzymatic hydrolysis of moth bean (*Vigna aconitifolia*) protein

**DOI:** 10.3389/fnut.2023.1167259

**Published:** 2023-06-09

**Authors:** Nancy Goyal, Sachin N. Hajare, Satyendra Gautam

**Affiliations:** ^1^Food Technology Division, Bhabha Atomic Research Centre, Mumbai, India; ^2^Life Sciences Department, Homi Bhabha National Institute, Mumbai, India

**Keywords:** *Vigna aconitifolia*, protein hydrolysate, antihypertensive peptide, ACE inhibition, molecular dynamics

## Abstract

**Aim:**

Dietary approaches for the regulation of blood pressure are the need of the hour. Hence, identifying the foods possessing such activity is gaining importance. With this aim, moth bean (Vigna aconitifolia), an underutilized pulse, was explored for the presence of antihypertensive activity in terms of angiotensin converting enzyme (ACE)-inhibition bioactivity.

**Methods:**

Defatted moth bean protein concentrate was hydrolyzed by using different proteases including Alcalase, papain, and trypsin, to identify the enzyme producing highly potent ACE inhibitory peptides. The hydrolysate showing the highest ACE inhibitory activity was further fractionated using an ultrafiltration membrane (10, 3 and 1 kDa) based on ACE inhibitory activity. The active fraction was further subjected to the ion-exchange chromatography followed by RP-HPLC and LC-MS/MS analysis for the enrichment and identification of ACE inhibitory peptides. Finally, based on the bioinformatic analysis, few peptides were synthesized and evaluated for ACE inhibitory activity, followed by docking study and molecular dynamic simulation of a peptide with the highest ACE inhibitory activity.

**Results and discussion:**

Out of the three proteases, Alcalase-derived hydrolysate showed the highest (~59%) ACE inhibition activity. Molecular weight-based fractionation revealed that <1 kDa fraction possessed the highest ACE inhibitory activity. Activity guided separation of 1 kDa fraction using ion-exchange chromatography, RP-HPLC and LC-MS/MS showed the presence of about 45 peptides. Based on the bioinformatic analysis, 15 peptides were synthesized and evaluated for ACE inhibitory activity. Among these, a novel octapeptide FPPPKVIQ showed the highest ACE inhibitory activity (93.4%) with an IC50 of 0.24 μM. This peptide retained about 59% activity post gastrointestinal digestion simulation. A Dixon plot as well as docking studies revealed the uncompetitive inhibitory nature of this peptide with a Ki value of 0.81 μM. Molecular dynamic simulation studies till 100 ns ensured the stability of the ACE-peptide complex.

**Conclusion:**

Thus, present study identified a novel potent ACE inhibitory peptide from moth bean that can be incorporated in a functional dietary formulation for regulation of hypertension.

## 1. Introduction

Hypertension or persistent high blood pressure is the most prevalent reason for the burden of cardiovascular diseases, all over the world. According to a study of the global burden of diseases, hypertension caused 1.63 million deaths in India in 2016. Blood pressure in mammals is majorly controlled by a cardiovascular regulatory system including the baroreceptor reflex, release of nitric oxide (NO) from the endothelium of blood vessels, the renin–angiotensin system (RAS), and the kallikrein–kinin system (KKS) (1). Among these, angiotensin-1 converting enzyme (ACE) is the key enzyme having multiple functions (2). Renin (produced by kidneys) converts inactive angiotensinogen (produced by the liver) to angiotensin 1 (Ang 1) which is further converted to Ang II by ACE. Ang II is a vasoconstrictor leading to elevated blood pressure. In addition to this, ACE can also hydrolyze bradykinin (a vasodilator) to an inactive product which reduces the ability of blood vessels to dilate. Due to these multiple activities, ACE inhibitors always constitute the first line of treatment for controlling hypertension. Moreover, there are other therapeutic agents available to treat hypertension, which includes diuretics, calcium antagonists, angiotensin II receptor antagonists, and α- and β-receptor blockers (3).

Different synthetic ACE-inhibitory drugs are available in the market, such as captopril, enalapril, and lisinopril. However, consistent therapy with these medicines has been correlated with a higher risk of adverse side effects, such as electrolyte disturbances, hypotension, syncope, and angioedema (4). Therefore, natural ACE inhibitors are believed to be a safe alternative to synthetic drugs. Natural compounds such as polyphenols (5), pigments (6), flavonoids (7), polysaccharides (8), alkaloids, tannins, peptides (9), and sterols (10) have been identified as ACE inhibitors. Among them, bioactive peptides got predominant attention because of their ability to perform a wide range of functions simultaneously, such as antihypertensive, antioxidant, antimicrobial, anticancer, anti-inflammatory, and immunomodulatory agents (11). Such biologically active peptides have been purified from many food sources, such as soybean, mung bean, milk, pea, rice, egg, and rapeseed proteins (12). These peptides have been generated either by enzymatic hydrolysis and fermentation or genetic recombination in bacteria and yeast (3) and were shown to possess significant antihypertensive activity. While investigating the mechanisms of these peptides, it was observed that ACE inhibition was the predominant mode of action. It has been shown that ACE-inhibitory peptides effectively decrease blood pressure in humans, without the accompanying adverse side effects (13). Therefore, many researchers have focused on producing antihypertensive peptides obtained from plant edible proteins due to the ease of preparation (14). The size of most ACE-inhibiting peptides was observed within a range of 2 to 20 amino acid residues (15).

With the aim to isolate and characterize a highly potent ACE-inhibitory molecule, we selected moth bean for the present study. Moth bean (*Vigna aconitifolia* L.), also known as matki (in the local language in India), is a drought-tolerant annual legume belonging to the family Fabaceae. It is grown mostly in dry regions of the country with scanty rainfall. Major moth bean-producing states in India include Rajasthan, Gujarat, Punjab, Haryana, Maharashtra, and Karnataka (16). Once considered an underutilized pulse, the moth bean is gaining importance due to the unavailability of conventional pulses owing to the supply–demand imbalance. This has led the researchers to evaluate the functional and pharmacological roles of moth bean cultivars. The seeds of moth bean are nutritionally rich containing ~23% protein, 62% carbohydrates, 1.6% fat, and various micronutrients including riboflavin, niacin, folate, and ascorbic acid (17). It is being observed that phenolic extracts of moth bean varieties possess antioxidant, antihypertensive, and anti-diabetic properties. This commodity has never been explored thoroughly for the presence of antihypertensive activity. Recently, Bhadkaria et al. (18) have reported small molecular weight peptides possessing ACE inhibition (~45%) activity, with IC_50_ value of 11.19 μg/ml. However, these peptides may not be stable in the gastrointestinal (GI) tract as they possessed chymotrypsin and pepsin cleavage sites. The stability of the peptides in the GI tract is one of the essential criteria required by such molecules.

The present study aimed at the purification and characterization of potent ACE-inhibitory peptides from moth bean protein hydrolysate. This was achieved by subjecting the defatted moth bean powder to different proteases, including Alcalase, papain, and trypsin. The hydrolysate giving maximum ACE inhibitory activity was further fractionated by using ultrafiltration, ion exchange chromatography, and reversed-phase high-performance liquid chromatography (RP-HPLC), to obtain a pool of ACE-inhibitory peptides. The sequence of individual peptides in the pool was identified using LC-MS/MS. The peptides were further screened *in silico* using various databases of antihypertensive peptides (AHTPDB and BIOPEP), along with enzymatic assays to identify the peptide with the highest ACE inhibitory activity. The gastrointestinal digestibility of these peptides was also studied to evaluate their stability in the gastrointestinal tract. Furthermore, inhibition kinetics of the most potent peptide was carried out to decipher the mechanism of ACE inhibition. Finally, molecular dynamics simulations, including docking studies, were carried out to confirm the mechanism of action and stability of the peptide-ACE complex.

## 2. Materials and methods

### 2.1. Materials, chemicals, and reagents

Moth bean seeds (MBS-27 variety) were obtained from Mahatma Phule Krishi Vidyapeeth (MPKV), Rahuri, Maharashtra. Angiotensin-converting enzyme (ACE) was extracted and purified from pig lung tissues according to the protocol described by Abdulazeez Mansurah et al. (19). Pig lung tissues were brought from a local slaughterhouse (Deonar Abattoir, Mumbai). Pepsin (1,000 U/mg, from porcine), pancreatin 3x (3 NF/USP, from porcine pancreas), trypsin (>2,000 U/g, from bovine pancreas), and papain (from papaya latex) were purchased from HiMedia Laboratories Pvt. Ltd., Mumbai. HHL (N-Hippuryl- His- Leu hydrate) and captopril were purchased from Sigma–Aldrich Chemicals Private Limited, Bangalore, India. All the chemicals used were of analytical grade.

### 2.2. Preparation of defatted moth bean protein powder (DMP)

Moth bean seeds (100 g) were ground to a fine powder and sieved through a 250-micron mesh. The powder was suspended in 500 ml of n-hexane (1:5, w/v) and stirred continuously for 2 h using a magnetic stirrer, followed by filtration through Whatman no. 1 filter paper to extract defatted moth bean powder (DMP). The procedure was repeated twice. The DMP obtained was dried in the oven at 45°C and stored at −20°C till it was utilized for further study.

### 2.3. Crude protein content determination

The crude protein content of the DMP was determined by estimation of the total nitrogen content by the Kjeldahl method, as described in AOAC international (20). The total protein content was calculated by multiplying the % nitrogen content obtained by a conversion factor of 6.25.

### 2.4. Preparation of protein hydrolysate

Defatted moth bean powder (DMP) of moth bean seeds was used for preparing protein hydrolysate using three different proteases, *viz.*, Alcalase, papain, and trypsin. To achieve this, DMP was suspended in distilled water (10% w/v), to prepare the solution to be used for the hydrolysis with the said enzymes. Enzyme and DMP were used in a ratio of 1:10 (w/w). DMP was hydrolyzed with Alcalase, papain, and trypsin at their optimum conditions, and fractions were collected at different time intervals of 1 h from 0 to 6 h. The optimum conditions of all three enzymes in terms of pH and temperature were standardized previously (data not shown), while to determine the optimum time for hydrolysis, reactions were performed till 6 h with sampling at each hour. During hydrolysis, the pH of the DMP solution was maintained at an optimum pH level by the addition of 2N NaOH. The conditions used for hydrolysis are shown in Supplementary Table S1. Fractions were heated at 90°C for 10 min to inactivate the enzymes. The supernatant was collected as moth protein hydrolysate (MPH) after centrifugation at 10,000 g at 4°C for 30 min. Fractions were stored at −20°C for further study.

### 2.5. Degree of hydrolysis (DH)

The DH of hydrolysates was determined by the Cd-ninhydrin method as described by Doi et al. (21) and Jiang et al. (22). In brief, the Cd-ninhydrin reagent was prepared by dissolving ninhydrin (0.4 g) and CdCl_2_ (1 g) in a solution containing 95% ethanol (80 ml), acetic acid (10 ml), and distilled water (20 ml). The sample solution (0.5 ml) was mixed with 1 ml of Cd-ninhydrin reagent and incubated for 10 min at 80°C in a water bath. Subsequently, the tubes were cooled on ice, and the absorbance was measured at 508 nm by a UV-VIS spectrophotometer. The total degree of hydrolysis or the total number of amino acid groups was determined by the complete hydrolysis of the sample using 6 N HCl at 110°C for 24 h. The free amino content in moth bean hydrolysates was expressed as Leucine equivalent, based on the standard curve generated using Leucine. The DH values were calculated using the following formula:


$$\begin{array}{l} \text{DH }\left(\%\right)=\frac{\left[\text{free amino groups after hydrolysis}\right]\text{−}\left[\text{free amino groups before hydrolysis}\right]}{\left[\text{total amino groups of moth bean protein}\right]\text{−}\left[\text{free amino groups before hydrolysis}\right]}\times 100\; \end{array}$$

Distilled water containing the same amount of proteases served as enzyme blank for all the hydrolysate samples. For calculating the degree of hydrolysis, enzyme blank readings were subtracted from the sample readings.

### 2.6. ACE inhibition activity determination

Angiotensin-converting enzyme (ACE) inhibition activity was performed according to Jimsheena et al. (23), with minor modifications. In brief, 10 μl (0.1 mg of peptides/ml) of the sample was first incubated with 10 μl of ACE at 37°C for 5 min. Later, 30 μl of 50 mM phosphate buffer (pH 8.3) containing 150 mM NaCl and 50 μl of 5 mM HHL was added and mixed completely. The reaction mixture was kept for incubation in a water bath at 37°C for 30 min. The reaction was terminated by adding 100 μl of 1 M HCl. Subsequently, 200 μl of pyridine and 100 μl of benzene sulfonyl chloride (BSC) were added sequentially to the solution and vortexed for 10 s. The reaction mixture was, then, cooled on ice, and the absorbance was measured at 410 nm using a spectrophotometer. Captopril was used as a reference for comparison. ACE inhibition activity (%) was calculated as follows:


$$\begin{array}{l} ACE\;inhibition\;\%\;=\;(1-\;As/Ac)\;x\;100\; \end{array}$$

where A_s_ is the absorbance of the mixture containing the sample, and A_c_ is the absorbance of the mixture without inhibitor at 410 nm. IC_50_ values were determined for each fraction using a plot of ACE activity vs. inhibitor concentration.

### 2.7. Fractionation of ACE-inhibitory peptides

#### 2.7.1. Ultrafiltration

The hydrolysate showing the highest ACE inhibition activity was fractionated sequentially (based on molecular weight) by using a high-pressure ultrafiltration device (Amicon^®^, Sigma–Aldrich Chemicals Pvt. Ltd., Bangalore, India), through ultrafiltration membranes having a molecular weight cutoff of 10, 3, and 1 kDa, respectively. The three fractions of MPH obtained were designated as F2 (<10 kDa), F3 (<3 kDa), and F4 (<1 kDa). The protein hydrolysate was designated as F1. These fractions were collected, freeze-dried, and stored at −20°C till further study.

#### 2.7.2. Ion exchange chromatography

The lyophilized fraction obtained after ultrafiltration was suspended in distilled water and loaded on an anion exchange column (DEAE Sepharose) that was equilibrated with 20 mM phosphate buffer (pH 8). The bound peptides were eluted with a gradient of NaCl (0 to 1 M) in 20 mM phosphate buffer (pH 8) at a flow rate of 1 ml/min. All the fractions were evaluated for the ACE inhibitory activity, and the active fraction was further loaded on CM Sepharose cation exchange column, pre-equilibrated with 20 mM acetate buffer (pH 4.5). Elution was performed by using a NaCl gradient (0 to 1 M) in 20 mM acetate buffer (pH 4.5). The unbound fraction of CM Sepharose (CM-UB) was also collected. All fractions were lyophilized and analyzed for ACE inhibition activity.

#### 2.7.3. Reversed-phase chromatography

The fraction of CM Sepharose (CM-UB) showing the highest ACE inhibitory activity was analyzed by reversed-phase high-performance liquid chromatography (RP-HPLC) using a Quaternary pump (Azura P6.1L, Knauer, Germany) and C_18_ Reverse Phase column (ODS Hypersil C_18_, Thermo Fisher Scientific, Switzerland). Mobile phase A consisted of deionized water containing 0.1% TFA while acetonitrile containing 0.1% TFA constituted mobile phase B. The lyophilized sample was reconstituted in distilled water, and 20 μl of this fraction was injected into the column. The elution was performed by using a linear gradient of 0 to 50% of solvent B in 30 min at a flow rate of 1 ml/min. The absorbance was monitored at 220 nm with a UV detector. All fractions were collected, vacuum evaporated, and stored at −20°C for further analysis. Each fraction was reconstituted in deionized water and evaluated for ACE inhibitory activity. The fraction showing the highest ACE inhibition activity was again subjected to RP-HPLC for further resolution of the peptides.

### 2.8. Peptide identification

The RP-HPLC fraction showed maximum ACE inhibitory effect was characterized by using LC-MS/MS (LC-MS facility, C-CAMP, Bangalore, India). The samples were analyzed on a Thermo Scientific Orbitrap Fusion Tribrid Mass Spectrometer coupled to a Thermo EASY-nanoLC (1,200 series) system. The samples were desalted using ZipTip, lyophilized, and dissolved in 20 μL of 2% acetonitrile/0.1% formic acid. Subsequently, the sample (10 μL) was injected into an LC system (EASY nLC 1200, Thermo Fisher Scientific, Walther, MA, United States) comprising of a C18 column (EASY SPRAY PEPMAP RSLC, 3 μm; 50 cm x 75 μm; 100 Å), with the flow rate of 300 nL/min. The solvent system consisted of buffer A-−0.1% formic acid in MilliQ water and buffer B-−80% acetonitrile with 0.1 % formic acid. The separation of peptides was achieved with 15% buffer B (0 min). This was followed by a linear gradient of buffer B from 25% to 60% (2 min−42 min), further raising the buffer B proportion to 95% at 44 min, and eluting all the bound peptides by maintaining it at 95% till 49 min. The elution of the peptides was monitored at 220 nm. In MS, ions were detected on the Orbitrap detector, with the scan range (m/z) 300–1,800, while in MS/MS, ion fragments were detected on the Ion Trap detector, with a mass scan range of 150–2,000. Data were analyzed using PEAKS Studio version 8.0 (Bioinformatics Solutions Inc., Canada).

### 2.9. Peptides screening by *in silico* approach and synthesis

All the peptides identified from LC-MS/MS were primarily screened using the database of antihypertensive peptides, AHTPDB (http://crdd.osdd.net/raghava/ahtpdb/), and bioactive peptides, BIOPEP (http://www.uwm.edu.pl/biochemia/index.php/pl/biopep), for the presence of similar ACE-inhibitory peptides. The peptides obtained were also screened by using Peptide Ranker (http://distilldeep.ucd.ie/PeptideRanker/), which predicts the probability of bioactivity of peptides by providing an activity score. The nature of each peptide was assessed by using PepDraw (http://pepdraw.com/), to predict the hydrophobicity value of peptides and ProtParam (https://web.expasy.org/protparam/). ToxinPred (https://webs.iiitd.edu.in/raghava/toxinpred/) was used to predict the toxicity of each peptide. Based on the results of all these analyses, peptides showing higher scores of ACE inhibitory activity were commercially synthesized by the solid phase synthesis method (GL Biochem Ltd, Shanghai), with >95% purity. The synthesized peptides were further evaluated for ACE inhibition activity.

### 2.10. Gastrointestinal digestion simulation of the peptides

The peptides synthesized were subjected to GI digestion simulation, as per the procedure described by Minekus et al. (24), with minor modifications. Bile juice was prepared according to Versantvoort et al. (25). Three steps of digestion (the mouth, stomach, and small intestine) were simulated, and the fluid was prepared to mimic physiological conditions. The composition of the simulated fluid is presented in Supplementary Table S2. The pH of the solution was adjusted by using 5 N NaOH and 6 N HCl. The digestion process was carried out using a stirring water bath at 37°C. Sampling was carried out after each step of digestion, and % ACE inhibition and degree of hydrolysis were calculated. Enzymes were inactivated by heating the fraction at 90°C for 10 min., followed by centrifugation at 10,000 g at 4°C for 15 min. The supernatant obtained was stored at −20°C for later study.

### 2.11. ACE inhibition kinetics study

The mechanism of ACE inhibition by a peptide showing the lowest IC_50_ value was identified by the rate of HHL hydrolysis measured over a range of concentrations (1 to 7.5 mM) by varying concentrations of inhibitory peptide (5 and 10 μM). The analysis was carried out by using a double reciprocal Lineweaver–Burk plot, to determine the mode of ACE inhibition, Michaelis–Menten constant (K_m_), and the maximum velocity (V_max_). The inhibitor constant (K_i_) was determined by the Dixon plot.

### 2.12. Molecular docking of ACE with the potent peptide and molecular dynamics simulation

Conformations of the most potent peptide obtained after LC-MS/MS analysis were predicted using a *De novo* peptide structure prediction tool PEP-FOLD 3 (26, 27). The best-fit model with the lowest sOPEP value was used for the docking study. The peptide structure model was initially processed with AutoDock Vina, and the output file was saved in .pdbqt format. The crystal structure of angiotensin-converting enzyme in complex with lisinopril (PDB ID 1086) was downloaded from the protein database bank (RCSB-PDB) (http://www.rcsb.org/pdb/home/home.do). The structure file was first processed in Pymol to remove the lisinopril and other non-bonded hetero molecules. Later, the file was processed in AutoDock Vina. All the water molecules present in the structure were removed, and polar hydrogen molecules were added. Subsequently, Kollman charges were also added to the protein molecule of ACE. After processing, the file was saved as a .pdbqt file. The docking study was performed using a best-fit model of a peptide and angiotensin-converting enzyme molecule. The coordinates to run docking were as follows: x 40.540; y 37.234; and z 43.569. The output was visualized using PyMOL version 2.3.4. To evaluate the molecular interactions of the peptide atoms and ACE molecule, the docking output file was analyzed by Discovery Studio Visualizer (Dassault Systèmes BIOVIA, Vélizy-Villacoublay, France).

For molecular dynamics simulation, GROMACS 2019.4 version with CHARMM36 force field was used by utilizing CGenFF software. TIP3P water molecules were used for the solvation of the protein-octapeptide complex inside a cubic box. The system charge was neutralized by replacing 13 water molecules with Na+ ions. Later, energy minimization of the neutralized system was carried out until the maximum force reached below 10 kJ/mol. Once energy minimization was achieved, the equilibration of the protein-ligand complex was carried out using two steps. First by restraining the position of ligand in coordination with the protein followed by application of group temperature coupling to minimize the variation due to the temperature differences. Subsequently, the system was equilibrated by performing two independent position-restrained molecular dynamic simulations. Initially, constant temperature constant volume (NVT) MD simulation was performed at 300 K using the V-rescale, modified Berendsen thermostat. Later, a 100 ps NPT MD simulation was carried out at 300 K and 1 bar. Eventually, using the standardized conditions, a molecular dynamics simulation for 100 ns was carried out. For constraining the bonds that are connecting hydrogen atoms, a faster LINCS algorithm (linear constraint solver) was used. Computation of long-range electrostatic interactions was performed using the particle mesh method developed by Paul Ewald. For the analysis of resulting trajectories, built-in tools of GROMACS were used.

### 2.13. Statistical analysis

All the experiments were performed in three replicates (*n* = 3), and data were represented as mean ± SD. Analysis of variance (ANOVA) was used for the statistical analysis of the data by using GraphPad Prism version 7.0 for Windows (GraphPad Software, San Diego, California United States). The multiple comparison test was performed by using the statistical hypothesis of Tukey's testing. The level of significant difference and confidence was set at *p* < 0.05 and 95%, respectively.

## 3. Results and discussion

### 3.1. Papain displayed the highest degree of hydrolysis of defatted moth bean powder

**Figure 1:**
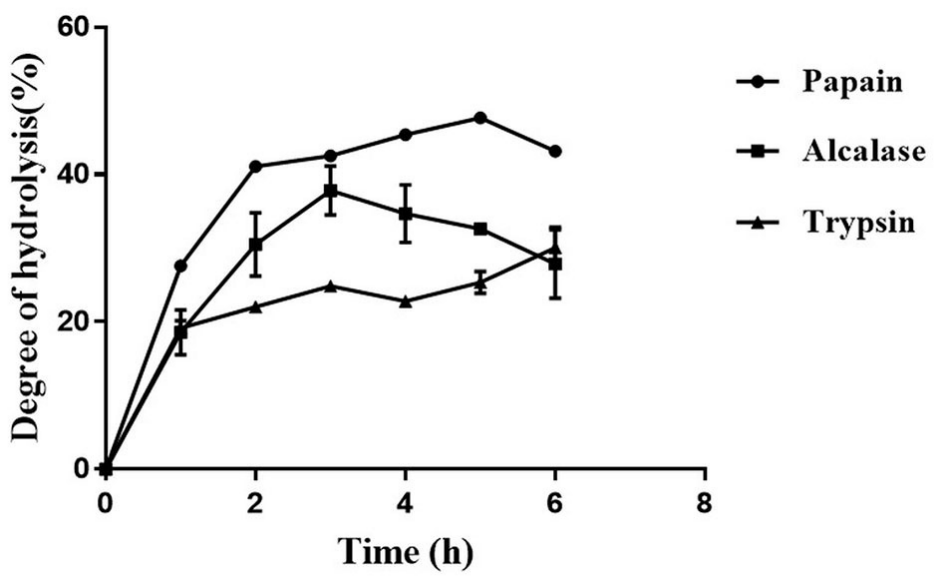
Temporal profile of degree of hydrolysis (%) of moth bean proteins following protease treatment. Data points are presented as the mean and standard deviation of the three replications.

The protein content in the MBS-2 variety of moth bean was 26.7 ± 0.8%, as estimated using a Kjeldahl method. The fat present in the seed powder was removed by extracting the powder with hexane. Defatted moth bean powder (DMP) was subjected to hydrolysis by three different proteolytic enzymes, to obtain a variety of peptides exhibiting ACE inhibition activity. The hydrolysis of proteins was examined by quantifying the degree of hydrolysis (DH), as shown in Figure 1. Enzyme papain showed the highest DH of 47.9 ± 0.9% at 5 h of hydrolysis, followed by Alcalase and trypsin hydrolysate with DH of 37 ± 3.3% and 30.1 ± 2.3% at 3 h and 6 h of hydrolysis, respectively. The highest DH was obtained by papain due to the broad specific nature of the activity. Papain hydrolyzes primarily the peptide bond at the C-terminal of lysine and arginine. Along with this, it also cleaves (minor activity) at the C-terminal of histidine, glycine, glutamic acid, glutamine, leucine, and tyrosine (28). On the contrary, Alcalase cleaves at the C-terminal of hydrophobic amino acids including alanine, methionine, valine, leucine, phenylalanine, proline, and tryptophan (29). When the amino acid content of moth bean proteins was studied, it was observed that the relative percentage of amino acids present at the cleavage site of papain is much higher (~32%) as compared with the amino acids present at the cleavage site of Alcalase (~10%) (30). This is depicted in the DH profile of papain and Alcalase hydrolysate. Trypsin is a serine endoprotease that cleaves specifically at the C-terminal of lysine and arginine residues only. Thus, the probability of the cleavage of proteins by trypsin during hydrolysis is much lower than that of the other two enzymes. Hence, the DH of trypsin was the lowest. There was a rapid increase in DH of defatted moth bean proteins during the 1st hour of hydrolysis, but subsequently, the rate of hydrolysis remained almost constant with a gradual increase (if any). The DH by Alcalase was maximum at 3 h of hydrolysis after which it decreased further. This decrease in DH can be attributed to different factors including enzyme inactivation, product inhibition of an enzyme, or depletion of the available hydrolysis site.

### 3.2. Highest ACE inhibition activity displayed by Alcalase-treated hydrolysate

**Figure 2:**
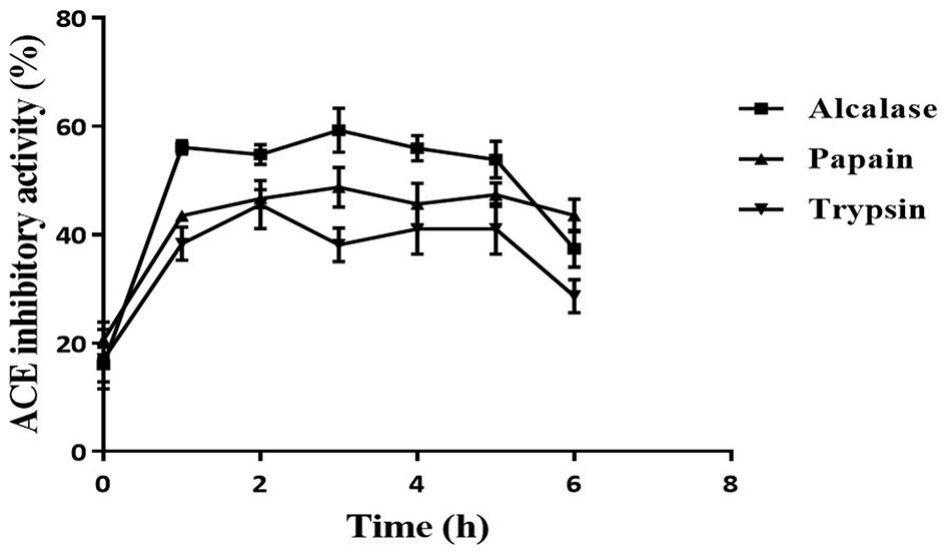
Effect of hydrolysis on ACE inhibition activity of defatted moth bean protein hydrolysate by proteases. Data points are presented as the mean and standard deviation of the three replications.

The ACE inhibition activity of different protease hydrolysates produced at specific time intervals (1 h) is presented in Figure 2. The unhydrolyzed fraction (0 h) showed approximately 15–20% inhibitory activity. This activity increased subsequently in all the samples when the proteins were subjected to the protease treatment. This increase can be attributed to the release of active peptides following the hydrolysis of proteins. The highest inhibitory activity of 59.2 ± 4.1% was shown by Alcalase hydrolysate at 3 h, followed by the papain (48.7 ± 3.7%) and trypsin hydrolysates (45.4 ± 4.4%). For all three proteases, the inhibitory activity increased till 3 h; thereafter, it plateaued. A similar pattern of ACE inhibition by mung bean protein hydrolysate was observed by Li et al. (31). Bhaskar et al. (32) also observed the highest ACE inhibitory activity at 3 h in Horse Gram protein hydrolysate prepared by digestion with Alcalase. Bhadkaria et al. (18), however, observed the highest activity in Alcalase-treated moth bean proteins within 30 min.

The DH differs for different enzymes because of their specificity toward the cleavage site, which leads to the production of various types of peptides. This is reflected in the ACE inhibition activity of the protein hydrolysates produced using different enzymes. Alcalase hydrolysate showed the highest ACE inhibition activity among all the proteases. In general, it is observed that peptides that contain hydrophobic amino acids possess higher ACE inhibitory activity (33). As seen earlier, Alcalase cleaves the proteins at the C-terminal of hydrophobic amino acids. This justifies the higher activity observed in Alcalase-treated hydrolysate. Based on the results, Alcalase hydrolysate was further fractionated for the isolation of ACE inhibitory peptides. Significant ACE inhibition activities of several food proteins following hydrolysis by Alcalase have been observed by many researchers (31, 34). It can be noted here that the unhydrolyzed fraction possessed very less activity (15%) that increased significantly following hydrolysis. Hence, it can be inferred that the peptides generated during hydrolysis were responsible for the ACE inhibitory activity. Moreover, in a separate study, the carbohydrates from moth bean seeds were extracted and evaluated for the ACE inhibitory activity. However, these carbohydrate molecules did not show ACE inhibitory activity (data not shown).

### 3.3. Enrichment of potent ACE-inhibitory peptides indicated the presence of small molecular weight and neutral/hydrophobic peptides

**Figure 3:**
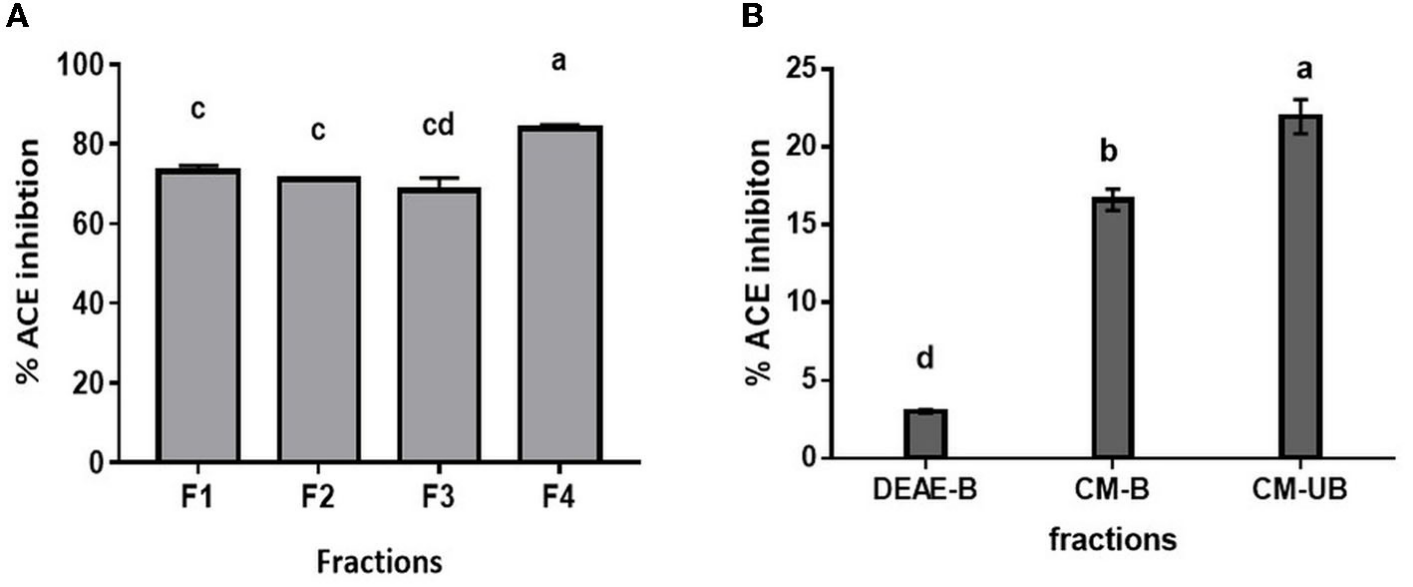
ACE inhibition profile of **(A)** ultrafiltrate fractions of Alcalase hydrolysate. **(B)** Ion-exchange fractions (F1, Alcalase hydrolysate; F2, <10 kDa; F3, <3 kDa; F4, <1 kDa; DEAE-B: DEAE bound fraction; CM-B, CM bound fraction; CM-UB, CM unbound fraction). Bars represent the mean ± standard deviation of the three replicates. ^a−*d*^Different letters across the columns depict that the mean values are significantly different at 0.05 level of significance (*p* ≤ 0.05) as analyzed by ANOVA.

Since Alcalase-treated hydrolysate exhibited the highest ACE inhibition activity, it was used for further fractionation. Based on the kinetic study of ACE hydrolysis and ACE inhibition, DMP was hydrolyzed with Alcalase for 3 h under optimum conditions. Subsequently, the hydrolysate was fractionated based on molecular weight using ultrafiltration membranes with a cutoff of 10, 3, and 1 kDa. All the fractions were evaluated for ACE inhibitory activity. As shown in Figure 3A, the 1 kDa filtrate fraction (F4) showed the highest ACE inhibition activity of 83.9 ± 0.9%. It has been reported that smaller peptides have higher ACE inhibition activity (33). It has been observed that the low molecular weight peptides show high ACE inhibition due to their high probability of interacting with the 3-D structure of ACE (35). To further enrich the ACE-inhibitory peptides, the 1 kDa fraction was subjected to ion exchange chromatography. Lyophilized 1 kDa fraction was first loaded on to DEAE Sepharose column and eluted with a salt gradient followed by the CM-Sepharose column. Unbound and bound fractions of both columns were evaluated for ACE inhibitory activity. As presented in Figure 3B, the bound fraction of the DEAE column (DEAE-B) showed 3.1 ± 0.1 % activity, indicating that anionic peptides did not have ACE inhibitory activity. CM-Sepharose bound fraction (CM-B) showed 16.6 ± 0.7% activity, indicating the partial cationic nature of some peptides showing ACE inhibitory activity. CM-unbound fraction (CM-UB) showed the highest inhibitory activity (21.4 ± 1.1%) suggesting that neutral or hydrophobic amino acid-containing peptides have the highest ACE inhibition activity (29).

**Figure 4:**
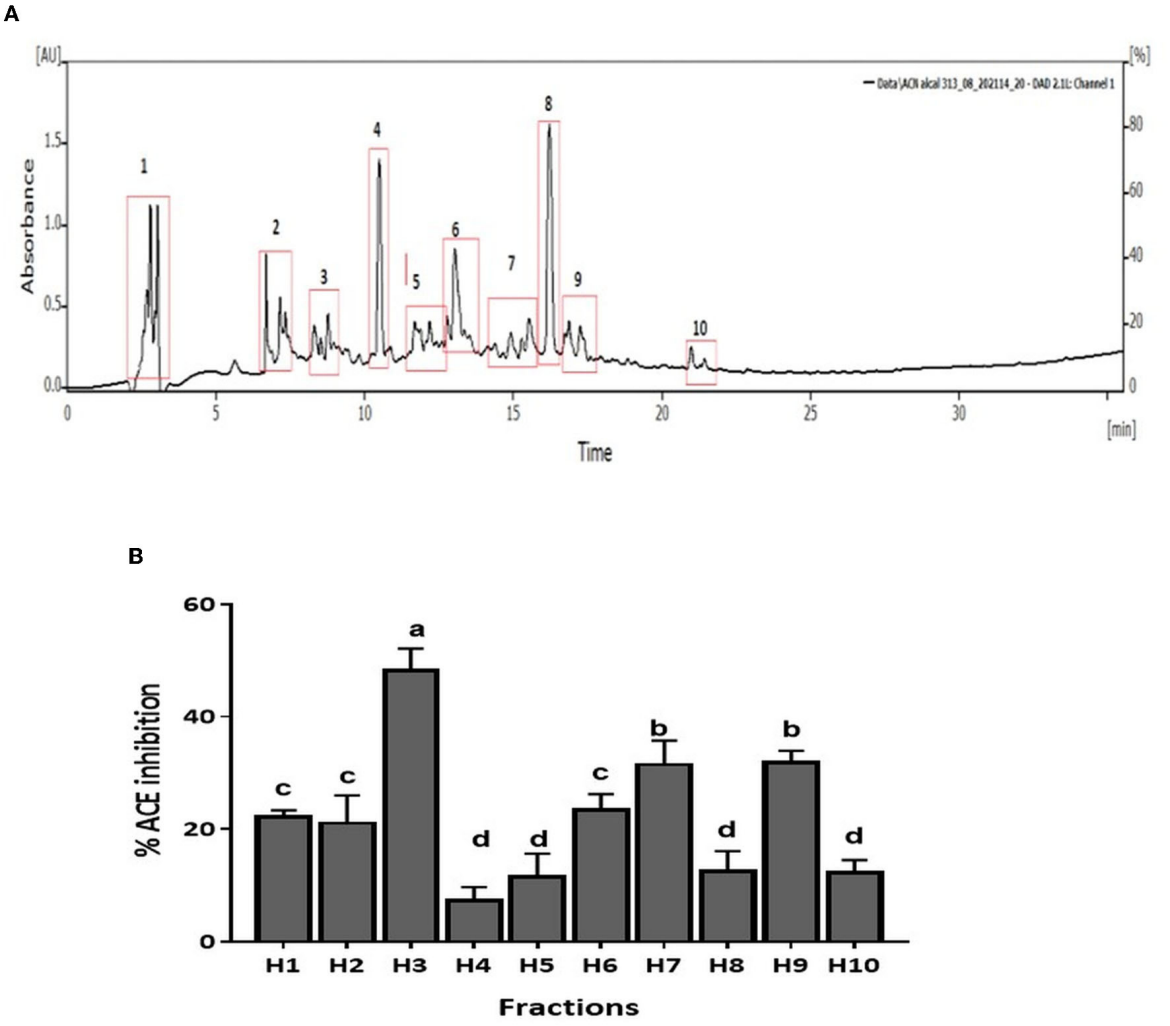
**(A)** RP-HPLC chromatogram of CM-UB fraction. **(B)** ACE inhibition (%) of fractions separated by RP-HPLC. Bars represent the mean ± standard deviation of the three replicates. ^a−*d*^Different letters across the columns depict that the mean values are significantly different at 0.05 level of significance (*p* ≤ 0.05) as analyzed by ANOVA.

**Figure 5:**
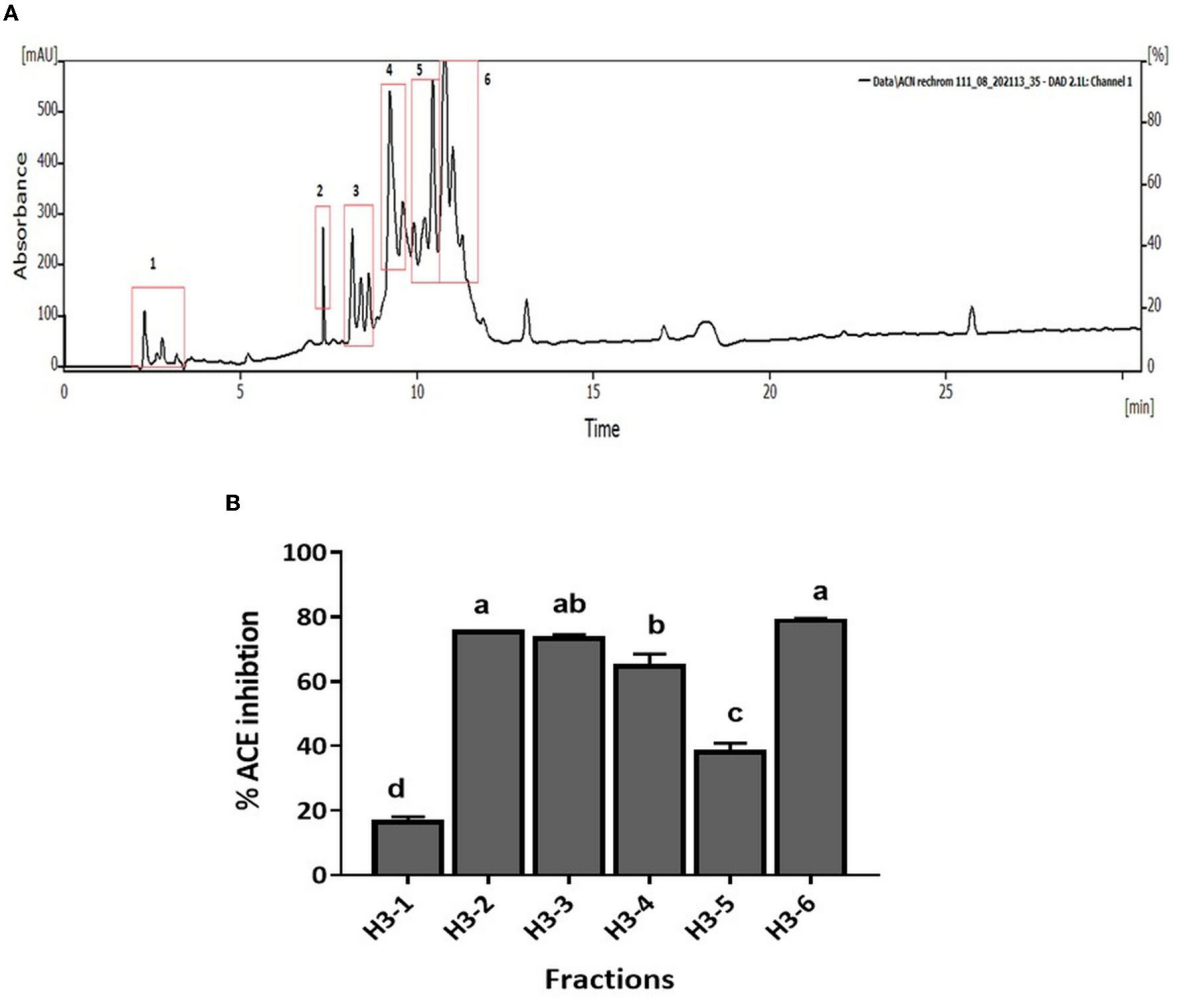
**(A)** RP-HPLC chromatogram of H3 fraction. **(B)** ACE inhibition (%) of fractions separated by RP-HPLC. Bars represent the mean ± standard deviation of the three replicates. ^a−*d*^Different letters across the columns depict that the mean values are significantly different at 0.05 level of significance (*p* ≤ 0.05) as analyzed by ANOVA.

CM unbound fraction possessing the highest ACE inhibitory activity was further resolved by RP-HPLC. The chromatogram profile showed multiple peaks over the entire run. The peaks eluted were collected in 10 fractions, and the ACE inhibition activity of these fractions was measured. Figure 4A shows the chromatogram of the RP-HPLC profile, where the peaks were divided into 10 fractions. All 10 fractions were, then, evaluated for ACE inhibitory activity (Figure 4B). As shown in Figure 4B, fraction H3 showed the highest ACE inhibition activity (48.5%), among all the fractions. H3 fraction was re-chromatographed for further purification on the same column, and the peptides eluted were collected in six fractions (H3-1 to H3-6) (Figure 5A). The ACE inhibitory activity of these six fractions was evaluated to identify the most potent fraction (Figure 5B). As shown in Figure 5B, the last fraction H3-6 showed the highest ACE inhibitory activity (~78.6%). Other fractions also showed considerable activity, indicating the presence of ACE-inhibitory peptides. However, for this study, the H3-6 fraction was selected, because of its high hydrophobic nature, for further analysis. Carbohydrate content analysis of this fraction revealed the absence of these molecules as these are high molecular weight moieties, and fractionation based on molecular weight had already eliminated them. This ruled out the possibility of the presence of the ACE inhibitory carbohydrate molecules in this fraction. Therefore, this fraction was further subjected to LC-MS/MS analysis to identify the number and sequence of all the peptides present in it.

### 3.4. Peptide identification in H3-6 fraction and screening of peptides by an *in silico* approach

LC-MS/MS indicated the presence of ~45 peptides of varying lengths in the H3-6 fraction. The sequence of peptides is shown in Table 1. These peptide sequences were used to predict the hydrophobicity index, and the predicted activity score using PepDraw and Peptide Ranker, respectively, is presented in Table 1. As shown in the Table, the hydrophobicity index ranged between 6.5 and 36.5 kcal/mol for the peptides pp-45 and pp-42, respectively. The predicted activity score for these peptides was in the range of 0.04 (pp-36, pp-37, and pp-32) to 0.99 (pp-42) (Table 1). The hydrophobicity index was an important parameter in determining the predicted ACE inhibitory activity of the isolated peptides. It was earlier reported that peptides with hydrophobic amino acids have a higher probability of possessing ACE inhibitory activity (36). Hence, this parameter was incorporated into this study. The Peptide Ranker software predicts the bioactivity of various peptides based on certain characteristics. This module was developed by training the neural networks using already known peptides possessing different bioactivities, such as antithrombotic activity, antimicrobial activity, anticancer, antioxidant, and immunomodulatory activities (37). Existing four databases of bioactive peptides, *viz.*, BIOPEP, APD2, PeptideDB, and CAMP were used by the researchers for the training of neural networks (37)). We used this tool to screen the peptides to predict their bioactivity scores. Based on the hydrophobicity index, predicted activity score, and the similarity of the peptides with the existing antihypertensive peptides in the databases (AHTPDB and BIOPEP), 15 peptides were synthesized (Table 2).

**Table 1:** The peptide sequence, hydrophobicity index [H index (kcal/mol)], and predicted activity score of identified peptides.

**Id**.	**Peptide sequence**	**H index (kcal/mol)**	**Predicted activity score**	**Id**.	**Peptide sequence**	**H index (kcal/mol)**	**Predicted activity score**
pp-1	GDTGRQQ	17.4	0.10	pp-24	NNPHR	13.8	0.44
pp-2	ADTGRQH	18.4	0.15	pp-25	AENNQR	16.3	0.09
pp-3	DTGRQH	17.9	0.12	pp-26	AFPAPGEKVE	18.2	0.34
pp-4	TGDTGRQH	19.3	0.09	pp-27	ATKPDAN	16.6	0.12
pp-5	TGDTGRQ	16.9	0.09	pp-28	FPPPKVIQ	8.6	0.53
pp-6	TGDTGR	16.2	0.14	pp-29	HPGDDKSG	23.2	0.30
pp-7	GDTGRQH	19.0	0.16	pp-30	HPGDDKS	22.6	0.20
pp-8	HDTGRQH	20.2	0.13	pp-31	AGKEEDKQ	26.8	0.05
pp-9	AGDTGRQH	19.5	0.21	pp-32	GKEEDKQD	30.0	0.04
pp-10	AGDTGRQ	17.2	0.23	pp-33	ATDETGSK	20.6	0.06
pp-11	KPDHRVE	21.8	0.15	pp-34	TDETGSKA	20.6	0.04
pp-12	KPSDSHQ	18.5	0.14	pp-35	SEGRGD	19.7	0.18
pp-13	NTGDEPLIT	15.4	0.14	pp-36	QQKQEQQQQQ	20.5	0.04
pp-14	GNPDIEHPE	22.3	0.28	pp-37	QKQEQQQQQ	19.7	0.04
pp-15	VEPLPPR	12.1	0.58	pp-38	LLTPAAGGN	9.9	0.21
pp-16	IEPLPPR	11.4	0.65	pp-39	ISGGSSR	12.3	0.19
pp-17	RPSKD	16.8	0.20	pp-40	VGHGGADG	18.5	0.20
pp-18	HVDSGK	17.8	0.11	pp-41	RGGSAGGRGGG	20.5	0.50
pp-19	HVDSGKS	18.3	0.10	pp-42	AGGGLGGGAGGGAGGGAGGGAGGGLGGGAGGGA	36.5	0.99
pp-20	VDSGKST	16.2	0.08	pp-43	GKGGGAGGGVGGGGGAG	26.2	0.79
pp-21	VNPDGRDSYIL	16.5	0.44	pp-44	LTAEHPVV	12.6	0.07
pp-22	NPDGRDS	19.6	0.18	pp-45	TFAPGF	6.5	0.91
pp-23	PDGRDS	18.7	0.21				

**Table 2:** Stability of synthesized peptides during GI digestion simulation after gastric (SGF) and intestinal (SIF) phase, measured by change in ACE inhibition activity.

**Id**.	**Peptide sequence**	**ACE inhibition % (before GI digestion)**	**ACE inhibition (%) after SGF**	**ACE inhibition (%) After SIF**
pp-1	GDTGRQQ	21.8 ±2.3^g^	24.0 ± 5.7^g^	7.6 ± 1.2^j^
pp-4	TGDTGRQH	16.5 ± 2.1^g^	18.0 ± 2.9^g^	33.6 ± 1.2^f^
pp-6	TGDTGR	3.9 ± 5.5^k^	3.9 ± 1.3^k^	4.2 ± 1.3^k^
pp-8	HDTGRQH	12.1 ±5.5^i^	11.8 ± 1.3^i^	21.3 ± 0.3^g^
pp-15	VEPLPPR	55.9 ± 1.3^c^	51.0 ± 3.4^c^	52.3 ± 3.8^c^
pp-16	IEPLPPR	36.0 ± 1.9^e^	30.0 ± 0.4^f^	33.6 ± 3.2^f^
pp-18	HVDSGK	8.2 ± 4.3^i^	9.6 ± 0.5^i^	15.3 ± 3.6^h^
pp-24	NNPHR	47.1 ±6.7^d^	41.4 ± 2.2^d^	15.0 ± 0.6^h^
pp-25	AENNQR	11.8 ± 2.9^i^	9.8 ± 2.8^i^	14.5 ± 0.5^h^
pp-28	FPPPKVIQ	93.4 ± 1.7^a^	90.5 ± 4.6^a^	59.6 ± 0.3^b^
pp-30	HPGDDKS	34.3 ± 7.7^e^	31.1 ± 0.7^e^	6.2 ± 4.3^j^
pp-33	ATDETGSK	13.1 ± 4.7^h^	15.1 ± 0.6^h^	15.6 ± 1.9^h^
pp-39	ISGGSSR	23.6 ± 6.9^g^	21.1 ± 0.8^g^	19.0 ± 1.8^g^
pp-44	LTAEHPVV	6.4 ± 1.0^j^	21.5 ± 3.4^g^	18.8 ± 9.9^g^
pp-45	TFAPGF	41.9 ± 6.9^d^	74.3 ± 2.2^b^	17.9 ± 1.8^g^

Where SGF and SIF are simulated gastric fluid and simulated intestinal fluid, respectively.

^a−*k*^Different letters across rows and columns depict that the mean values are significantly different at 0.05 level of significance (*p* ≤ 0.05) as analyzed by ANOVA.

### 3.5. *In vitro* gastrointestinal digestion simulation (IVGD) showed alteration in the ACE inhibitory activity of the peptides

The synthesized peptides were subjected to IVGD to simulate the conditions in the human alimentary canal if they were administered orally. The effect of this simulation was studied on the ACE inhibition activity of all these peptides as the GI digestion proceeds. As presented in Table 2, the ACE inhibitory activities of nine peptides changed after IVGD. Peptide pp-4 showed a significant increase (103%) in the ACE inhibitory activity after intestinal digestion simulation. This can be attributed to the cleavage at arginine by trypsin. Similarly, peptide pp-18 showed a significant increase (86%) in the ACE inhibitory activity following intestinal digestion simulation due to the cleavage of His at the first position by chymotrypsin. At the same time, peptide NNPHR (pp-24) showed a significant reduction (68%) in the ACE inhibitory activity due to the cleavage of His at the fourth position by chymotrypsin. Peptides pp-44 and pp-45 showed a significant increase (236% and 77%, respectively) in ACE inhibitory activity due to the cleavage of Leu and Thr by pepsin in these peptides. However, after IVGD, the activity decreased slightly in both of these peptides. In the case of peptides pp-1 and pp-30, the ACE inhibitory activity decreased significantly (to 35% and 18%, respectively) following intestinal digestion simulation due to the scission of Arg and Lys by trypsin in these peptides, respectively. The activity of peptide pp-28 (FPPPKVIQ), which exhibited the highest ACE inhibition, remained intact (90.5 ± 4.6%) after the gastric phase (i.e., resistant to pepsin digestion) but activity decreased (59.6 ± 0.3%) after the intestinal phase (Table 2). This is because the peptide lacks the pepsin cleavage site while it has a Lys at the fifth position, which is cleaved by the enzyme trypsin present in the intestine. Salampessy et al. (38) also reported that the peptide derived from the sunflower remains unchanged after pepsin digestion. Xu et al. (3) also reported the changes in the ACE inhibition of all the peptides from soybean protein isolate after GI digestion. Despite digestion by trypsin, the ACE inhibition of FPPPKVIQ (IC_50_: 20 μM) was found to be the highest among all the other peptides studied (59.6%). Therefore, further studies were carried out with this peptide. It can be noted that the peptide FPPPKVIQ exhibited significantly higher ACE inhibition activity (in terms of % inhibition and IC_50_ values) as compared with the peptides reported by Bhadkaria et al. (18). Moreover, the researchers have not studied the gastrointestinal stability of the peptides they have isolated and characterized. These peptides also showed the presence of chymotrypsin and pepsin cleavage sites when analyzed using Expasy online PeptideCutter tool. To ensure that the peptide FPPPKVIQ is not generated due to the cleavage/hydrolysis of the enzymes Alcalase, papain, and trypsin used in the preparation of hydrolysate, a similar search of the peptide sequence was carried out with all three enzymes (Supplementary Figure S1). The results showed no sequence similarity of the characterized peptide with any of the enzymes used, indicating that these enzymes were not the sources of the peptide (Supplementary Figure S1). Later, when the peptide FPPPKVIQ was blasted for sequence similarity with the genus *Vigna*, it showed the presence of this peptide in a linoleate 9S-lipoxygenase-like protein in a few other species of *Vigna* (including *V. radiata, V. unguiculata*, and *V. umbellate*) (Supplementary material 2). This protein is involved in various physiological processes in plants, including growth, development, and pest resistance. The protein BLAST (BLASTP) search of the peptide with *V. aconitifolia* revealed ~57% sequence homology of the peptide with non-TIR-NBS-LRR disease-resistant protein (Supplementary Figure S2). It can be noted that the complete genome sequence of the species *Vigna aconitifolia* is presently unavailable in the database, making it difficult to prove that the current peptide is derived from *V. aconitifolia*. However, the presence of this peptide in a number of other *Vigna* species supports our hypothesis (Supplementary material 2). The peptide showed no similarity in humans. A truncated peptide (FPPPKV) was found in the immunoglobulin heavy chain junction region.

### 3.6. Kinetics of the most potent ACE inhibitory peptide pp-28 revealed the uncompetitive nature of inhibition

**Figure 6:**
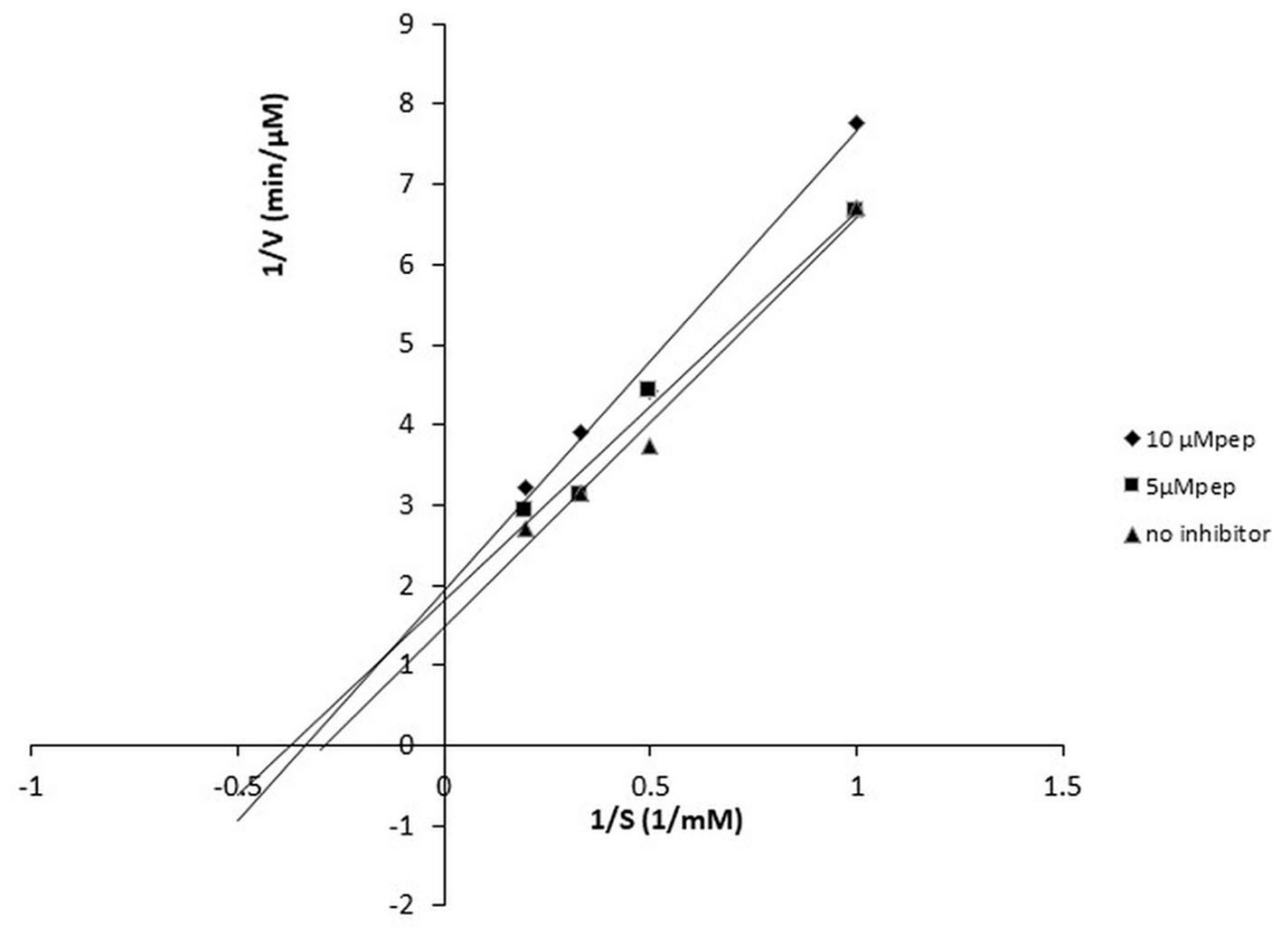
Lineweaver–Burk plot of ACE-inhibitory peptide FPPPKVIQ at different peptide concentrations.

The peptide FPPPKVIQ showed the highest ACE inhibition and was selected for further studies. The ACE inhibition pattern with FPPPKVIQ peptide was studied using the Lineweaver–Burk plot (Figure 6). Based on the plot, it can be concluded that the molecule is working as an uncompetitive inhibitor of ACE, indicating that the peptide has an ability to bind with ACE-substrate complex, to generate a dead-end complex. The V_max_ and K_m_ of the enzyme without inhibitor were 0.75 μM/min and 5.6 μM/min, respectively. The V_max_ and K_m_ values decreased in the presence of an inhibitor (0.4 μM/min and 3.35 μM/min, respectively). The Ki value of the peptide was observed to be 0.81 μM, as obtained by the Dixon plot. It has been reported earlier that short peptides containing phenylalanine, valine, isoleucine, and lysine have a higher probability of binding to ACE (29). The present peptide satisfies these criteria and is able to inhibit ACE with higher efficiency.

Fold purification of the ACE inhibitory peptides was evaluated during the entire process of peptide purification from Alcalase hydrolysate (Table 3). As shown in Table, re-chromatography of the HPLC-purified fraction increased the fold purification significantly (13.5), and the final fold purification of the peptide FPPPKVIQ was 36.6 as compared with that of the Alcalase hydrolysate. The final yield of the peptide obtained was 0.05% of the hydrolysate fraction.

**Table 3:** Purification fold of ACE-inhibitory peptide.

**Purification step**	**IC_50_ (μg/ml)**	**Purification fold**
Alcalase hydrolysate (MPH)	13.6	1
Ultrafiltration (<1 kDa)	11.9	1.1
Ion-exchange (CM-UB)	4.7	2.9
RP-HPLC (H3)	4.1	3.3
Re RP-HPLC (H3-6)	1.0	13.5
FPPPKVIQ	0.2	36.6

### 3.7. Molecular docking of octa peptide, pp-28, showed binding other than the active site

**Figure 7:**
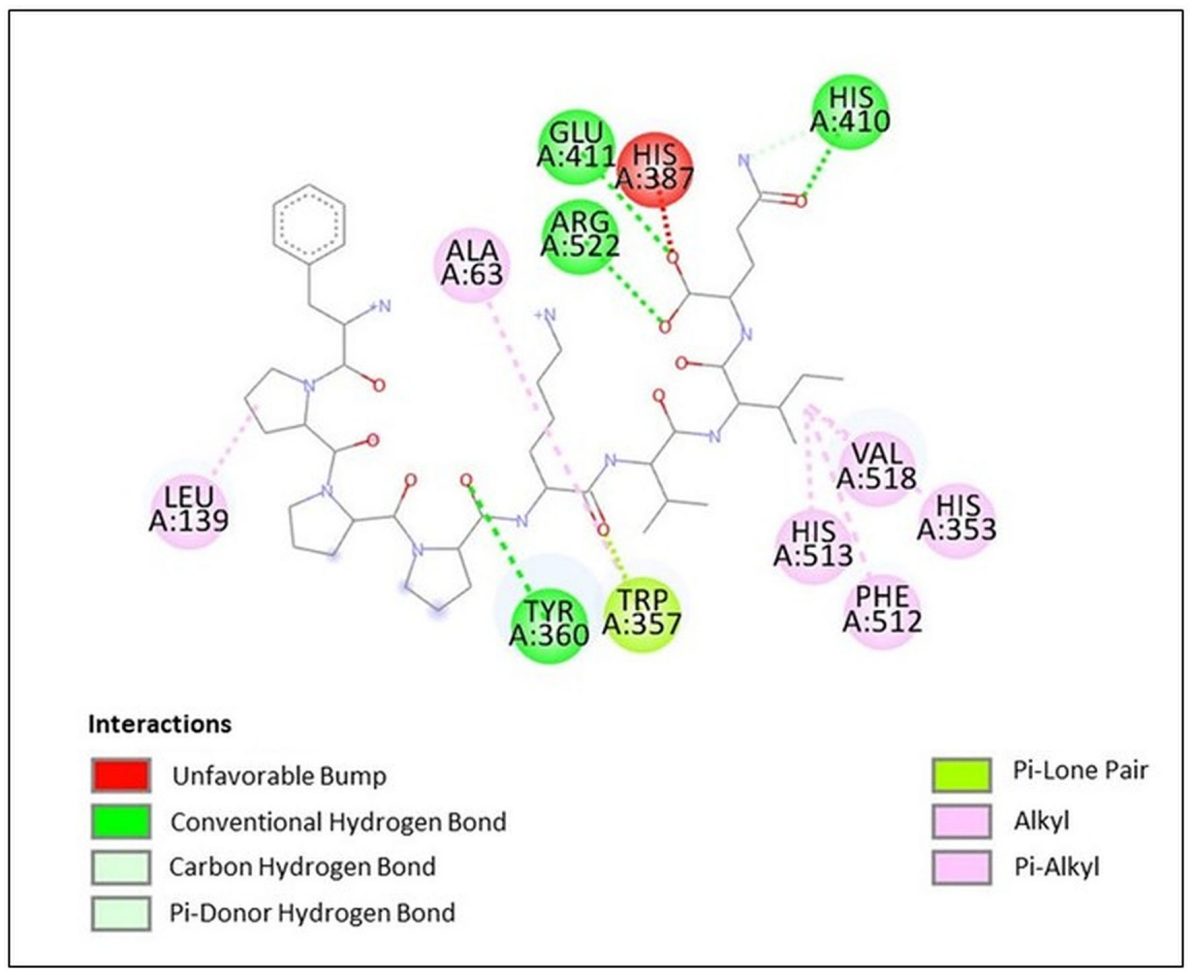
Docking results of peptide FPPPKVIQ with ACE molecule showing the interaction of the residues of ACE with peptide.

Docking study results in the peptide FPPPKVIQ with the ACE enzyme showed a binding energy (ΔG) value of −9.6 kcal/mol, RMSD of 25.26, and calculated Ki of 0.2 μM. In addition, the Dixon plot results indicated an uncompetitive mode of inhibition by the peptide. Hence, the docking results were analyzed using the Discovery Studio Visualizer to understand the binding site and the interacting residues of peptides with the ACE molecule. As shown in Figure 7, the peptide is found to form hydrogen bonds with the amino acid residues Tyr 360, His 410, Glu 411, and Arg 522 of the ACE molecule. Due to the hydrogen bonding of Glu 411 with the peptide molecule, an unfavorable bump has been created at His 387 by bending this residue in the ACE molecule. Other interactions including Van Der Waal, Pi-lone pair, Pi–Alkyl, and Alkyl–Alkyl helped to stabilize the interaction/binding of the peptide with the ACE molecule (Figure 7). In addition to other residues, the active site of the ACE molecule involves amino acid residues Tyr 224, His 383, His 387, Glu 411, Trp 485, Arg 489, and Arg 522 (39). His 383 and His 387 provide a site for the co-ordination binding of Zn^+^ with these residues, while additional coordination is provided by Glu 411. Arg 522 provides a site for Cl^−^ binding, which is important for the catalytic activity of ACE (39). Thus, in the absence of a substrate, when the peptide is bound to the ACE molecule, His 387 and Glu 411 become unavailable to provide coordination with the Zn^+^ atom. Additionally, Arg 522 is also occupied and becomes inaccessible to provide a site for Cl^−^ binding. This results in a significant reduction in the binding of the substrate to the catalytic site of ACE, subsequently decreasing its activity. Thus, the docking results re-confirmed the outcome of the Dixon plot that the peptide is binding to a site other than the active site.

### 3.8. Molecular dynamics simulation of most potent peptide pp-28 with ACE

**Figure 8:**
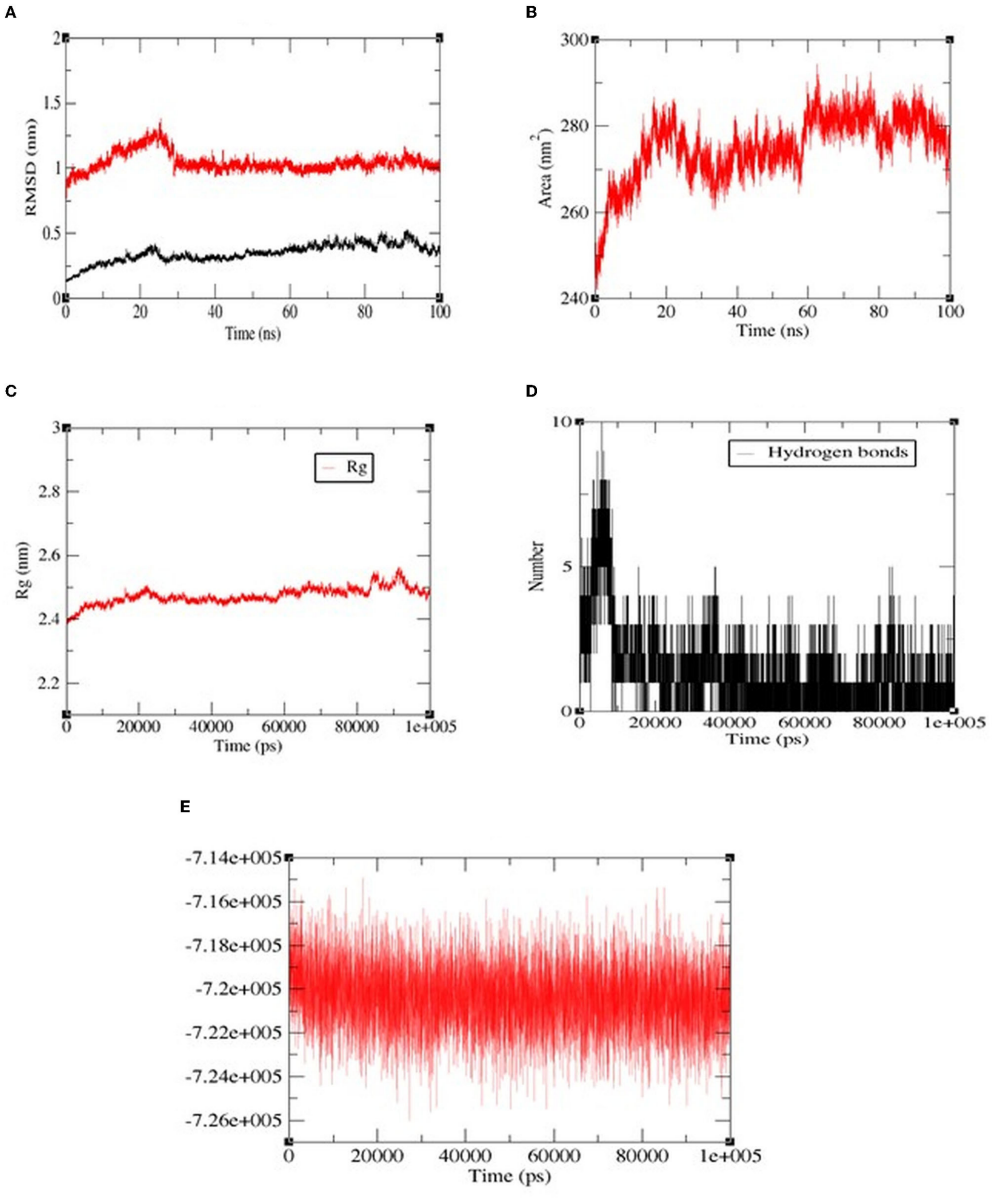
The molecular dynamics (100 ns) results of peptide FPPPKVIQ and the ACE complex. **(A)** Root mean square deviations (RMSD, nm), **(B)** Solvent accessible surface area (SASA) values, **(C)** Radius of gyration (Rg), **(D)** H-bond number, **(E)** The fluctuation of binding free energy of the complex.

Among all the peptides, pp-28 (FPPPKVIQ) showed the highest ACE inhibition activity. Hence, the stability of the peptide-ACE complex was studied by assessing conformational fluctuation (RMSD and SASA), compactness (R_g_), H-bond number, and energy changes under physiological conditions by molecular dynamics simulation (MDS). The MDS was carried out for the duration of 100 ns. RMSD analysis of the backbone atoms of the native protein and protein-ligand complexes was carried out to monitor the structural analysis of the complex. Figure 8A reveals the RMSD plot for native ACE and bound peptide (protein-ligand complex) for the 100 ns trajectory. From the data, it can be observed that native and protein-ligand complexes achieved equilibrium after 25 ns. The plot exhibited RMSD values of ACE protein (black) and complex (red) were 0.25 nm and 0.98 nm, respectively, throughout the time of interaction. This is acceptable and shows the stability of the complex. The solvent accessible surface area (SASA) of the complex (Figure 8B) increased initially to 20 ns, finally reaching equilibrium at 286 nm^2^ at 100 ns. The radius of gyration (R_g_) specifies the stability of the complex by calculating the structural compactness of biomolecules. As presented in Figure 8C, the R_g_ showed a relatively consistent value throughout the trajectory of the MDS, indicating it to be a stably folded structure. The number of H bonds (Figure 8D) between FPPPKVIQ and ACE increased from 6 to 10 during 20 ns. These bonds decreased further to 4–5 during the rest of the time till 100 ns of MDS. In terms of total energy, the complex achieved a stable state with the lowest energy from the beginning itself (Figure 8E).

All the above results have shown that the octapeptide has a potential ACE inhibitory activity which is stable to the gastrointestinal digestion simulation. Animal studies using hypertensive rats, if positive, would confirm its *in vivo* antihypertensive activity. Future studies can be directed toward the exploration of its pharmacological use. Currently, the process for the production of synthetic antihypertensive molecules is well optimized, due to which the production cost is significantly low. Bioactive peptides, though they possess great pharmacological potential, have limited applicability due to their high production costs. However, many researchers have been working on the reduction of the cost of peptide production, that includes use of a continuous manufacturing process, use of the μLOT technology platform, scaling up the process at a 5-liter scale, developing an autoinduction strategy for the expression of recombinant peptides, and increasing the production scale from 100 to 1,000 mg/batch (40). These strategies have reduced the cost of peptide production by approximately six times and made the price comparable to the chemical synthesis approaches (40). Thus, bioactive peptides may find a place in the market for synthetic drugs in the near future.

## 4. Conclusion

In the present study, moth bean proteins were explored for the presence of novel ACE inhibitory peptides following treatment with different proteases including Alcalase, papain, and trypsin. Based on the molecular weight fractionation, followed by a series of chromatographic techniques, a potent ACE inhibitory peptide was isolated and purified from the Alcalase hydrolysate of moth bean proteins. LC-MS/MS analysis confirmed the sequence of the novel octapeptide as FPPPKVIQ. The peptide retained significant activity (IC_50_ 20 μM) when subjected to the gastrointestinal digestion simulation. The results of the Dixon plot and docking studies revealed an uncompetitive binding of the peptide to the ACE molecule. Furthermore, molecular dynamics simulation studies, including RMSD, Rg, and number of hydrogen bonds, revealed the stability of the peptide-ACE complex. These results identified the novel octapeptide (FPPPKVIQ) as a potential functional prophylactic molecule. This peptide finds its application in the functional nutraceutical formulation, intended for the control of hypertension. *In vivo* studies of the peptide using the hypertensive rat model will have to be accomplished to ascertain its blood pressure-lowering efficacy.

## Data availability statement

The original contributions presented in the study are included in the article/Supplementary material, further inquiries can be directed to the corresponding author.

## Author contributions

NG carried out the experimental work, collected and analyzed the data, and drafted the manuscript. SH was involved in conceiving and designing the research project, critically analyzing and interpreting the data, and revising the manuscript thoroughly. SG was involved in overall monitoring of the progress of the work, critical suggestions during the experimental work, and revising the manuscript. All authors contributed to the article and approved the submitted version.
