# Supplementary material for: Release of an encrypted, highly potent ACE-inhibitory peptide by enzymatic hydrolysis of moth bean (Vigna aconitifolia) protein
Source: Front Nutr. 2023 Jun 9;10:1167259. doi: 10.3389/fnut.2023.1167259 (PMC10288869; doi:10.3389/fnut.2023.1167259)

Supplementary Material

**Release of an encrypted, highly potent ACE inhibitory peptide by enzymatic hydrolysis of moth bean protein**

**Nancy Goyal, Sachin N. Hajare^*^ and S. Gautam**

*** Correspondence:** Corresponding Author: shajare@barc.gov.in

**Table S1. Reaction conditions used for hydrolysis of defatted moth bean powder (DMP) with different enzymes.**

| Enzymes | Temperature | pH | Enzyme:DMP Ratio |
| --- | --- | --- | --- |
| Alcalase | 55 °C | 8.2±0.2 | (1:10) v/v |
| Papain | 50 °C | 7.0±0.5 | (1:10) w/v |
| Trypsin | 45 C | 8.5±0.2 | (1:10) w/v |

**Table S2. Chemical composition, protease concentration, and pH conditions of different fluids used for simulated gastrointestinal digestion.**

|  | **Salivary fluid** | **Gastric fluid** | **Intestinal fluid** | **Bile juice** |
| --- | --- | --- | --- | --- |
| **Chemical composition** | KCl (15.1mM)  NaHCO_3_ (13.6mM)  KH_2_PO_4_(3.7mM)  MgCl_2_(H_2_O)_6_(0.15mM)  (NH_4_)_2_CO_3_(0.06mM)  ^*^CaCl_2_(H_2_O)_2_ (0.75mM) | KCl(6.9mM)  NaCl(47.2mM)  NaHCO_3_(25mM)  KH_2_PO_4_(0.9mM)  MgCl_2_(H_2_O)_6_(0.1mM)  (NH_4_)_2_CO_3_(0.5mM)  ^*^CaCl_2_(H_2_O)_2_ (0.075mM) | KCl(6.8mM)  NaCl(38.4mM)  NaHCO_3_(85mM)  KH_2_PO_4_(0.8mM)  MgCl_2_(H_2_O)_6_(0.33mM)  ^*^CaCl_2_(H_2_O)_2_ (0.3mM) | KCl (2.5mM)  NaCl (60mM)  NaHCO_3_(3.4mM)  HCl (10mM)  Urea (80µM) |
| Enzyme | Diastase (75U/ml) | Pepsin (2,000U/ml) | Pancreatin (100U/ml) |  |
| pH | 7 | 2.5 | 8 |  |
| Time | 2 min. | 2 h | 2 h |  |

* CaCl_2_(H_2_O)_2_ is added to the final mixture of simulated digestion fluid to avoid precipitation.

**Figure S1. Sequence similarity search of the characterized peptide (FPPPKVIQ) with A) Alcalase B) Papain C) Trypsin.**

**(A)**


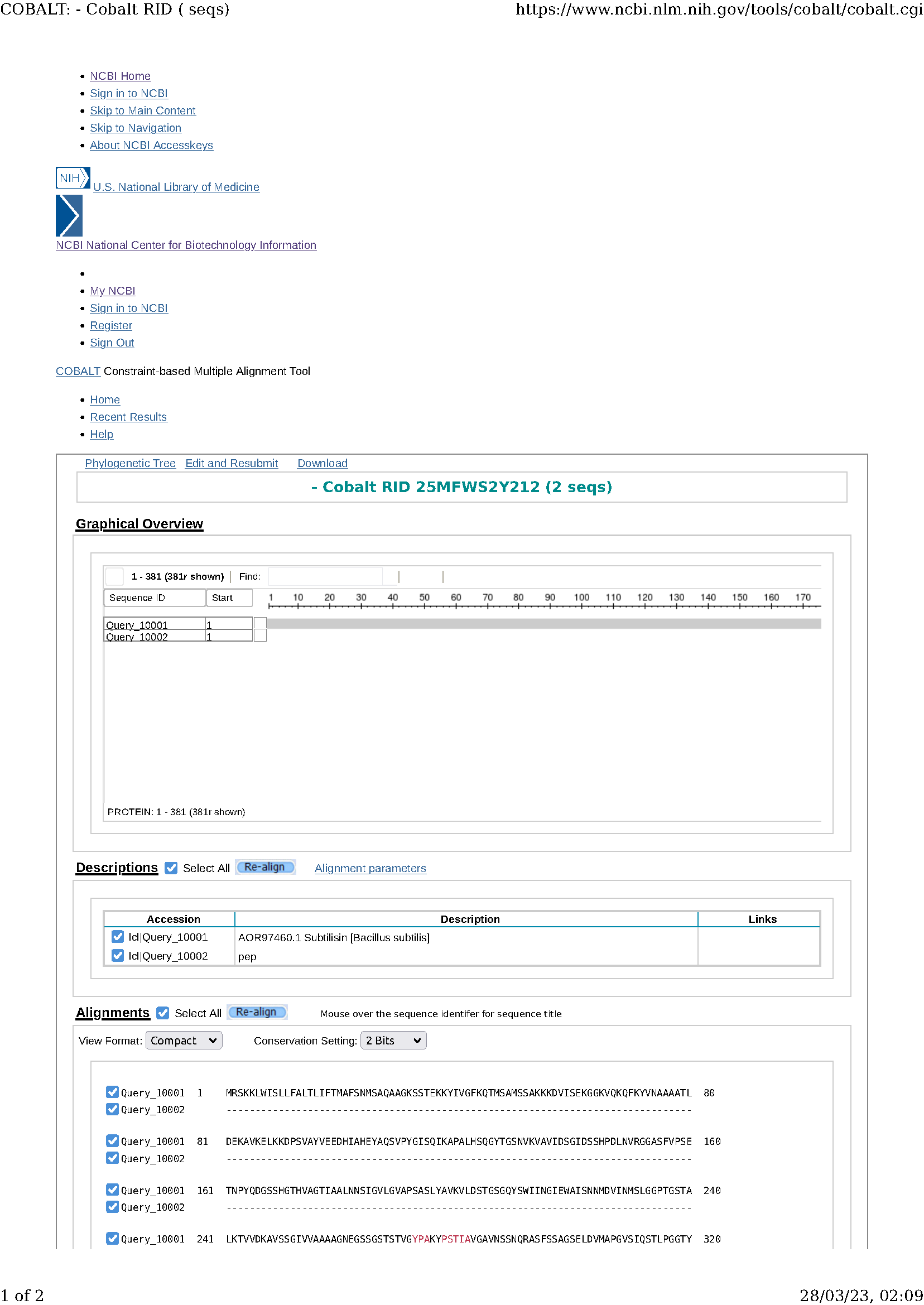


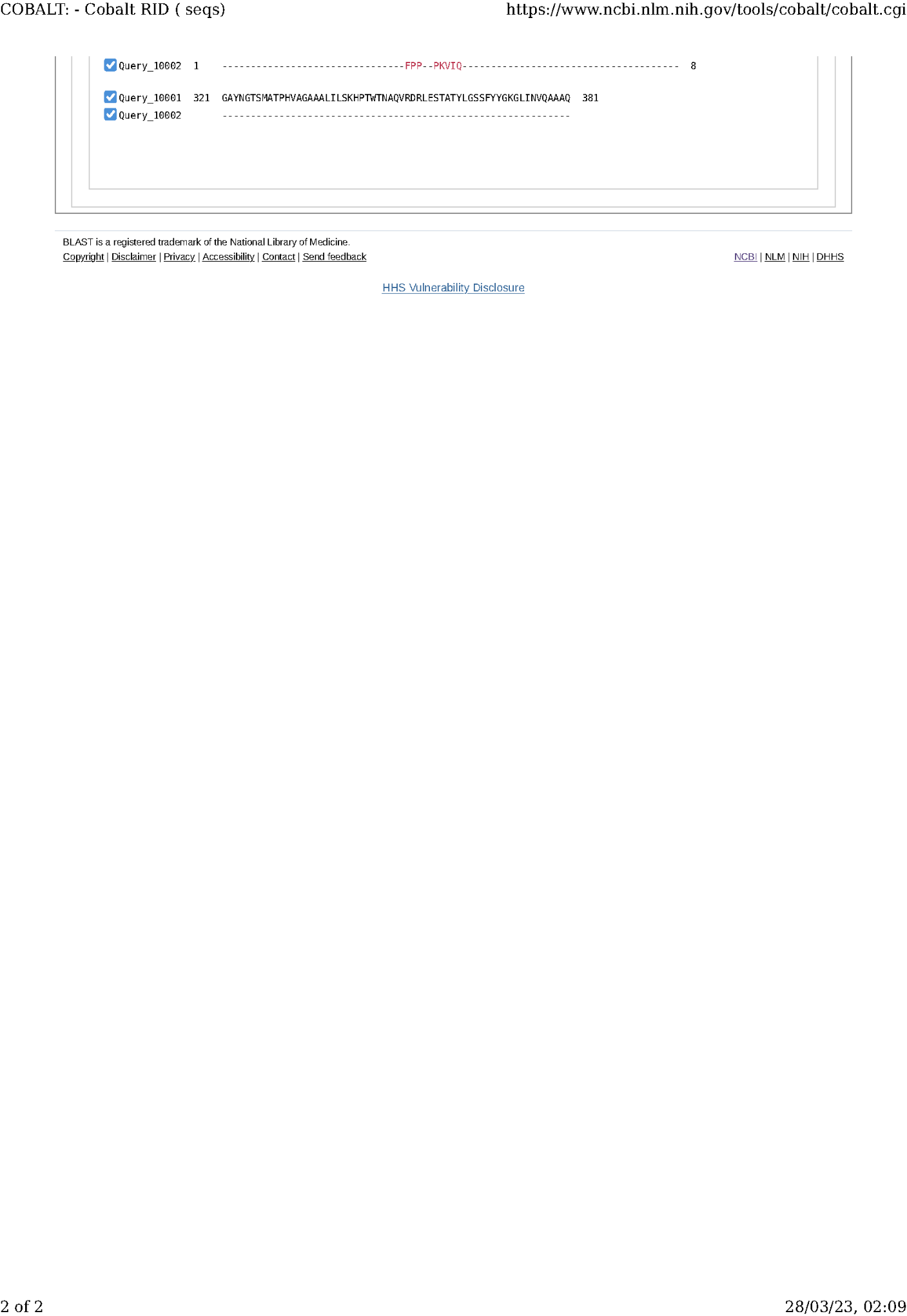


**(B)**


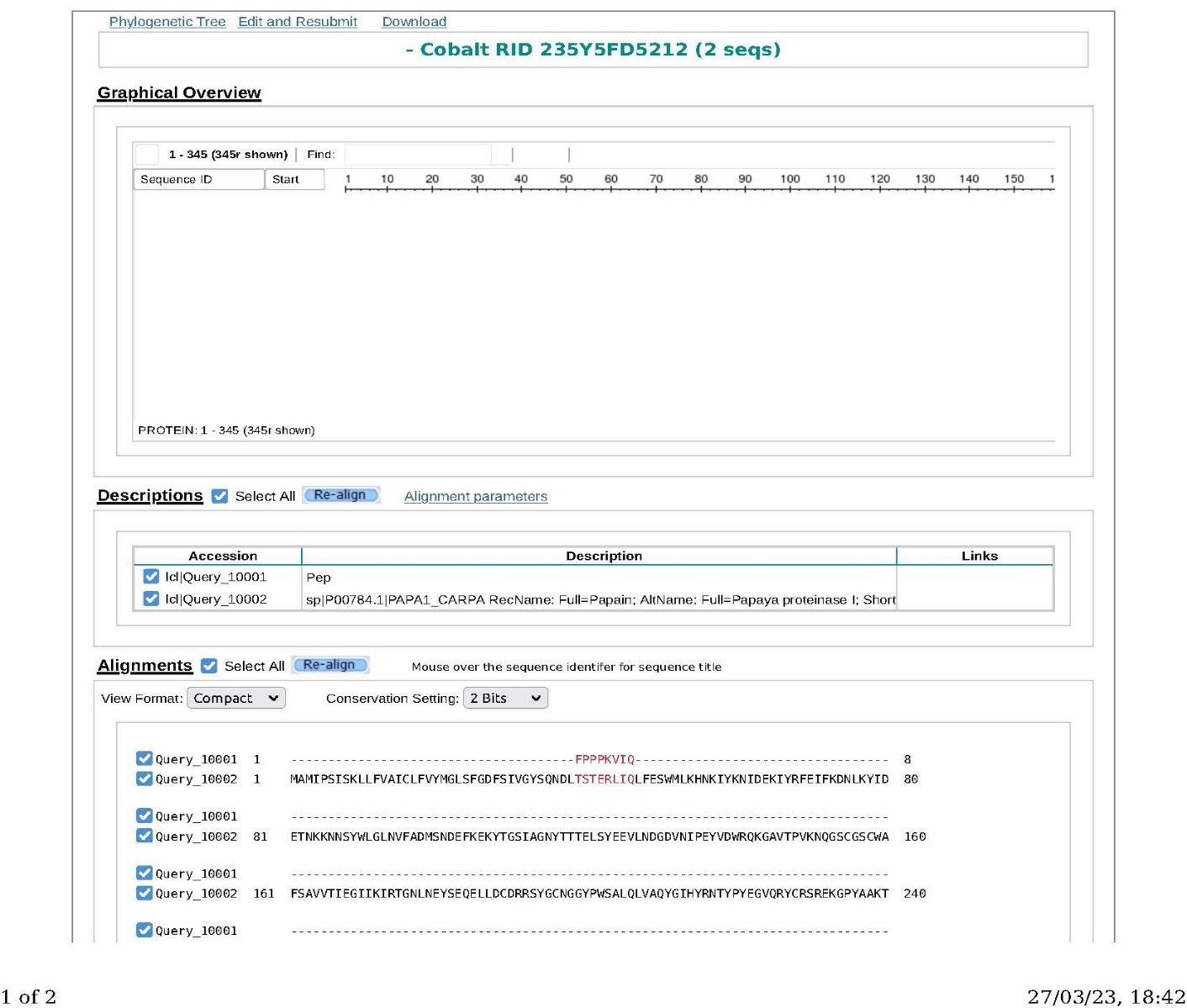


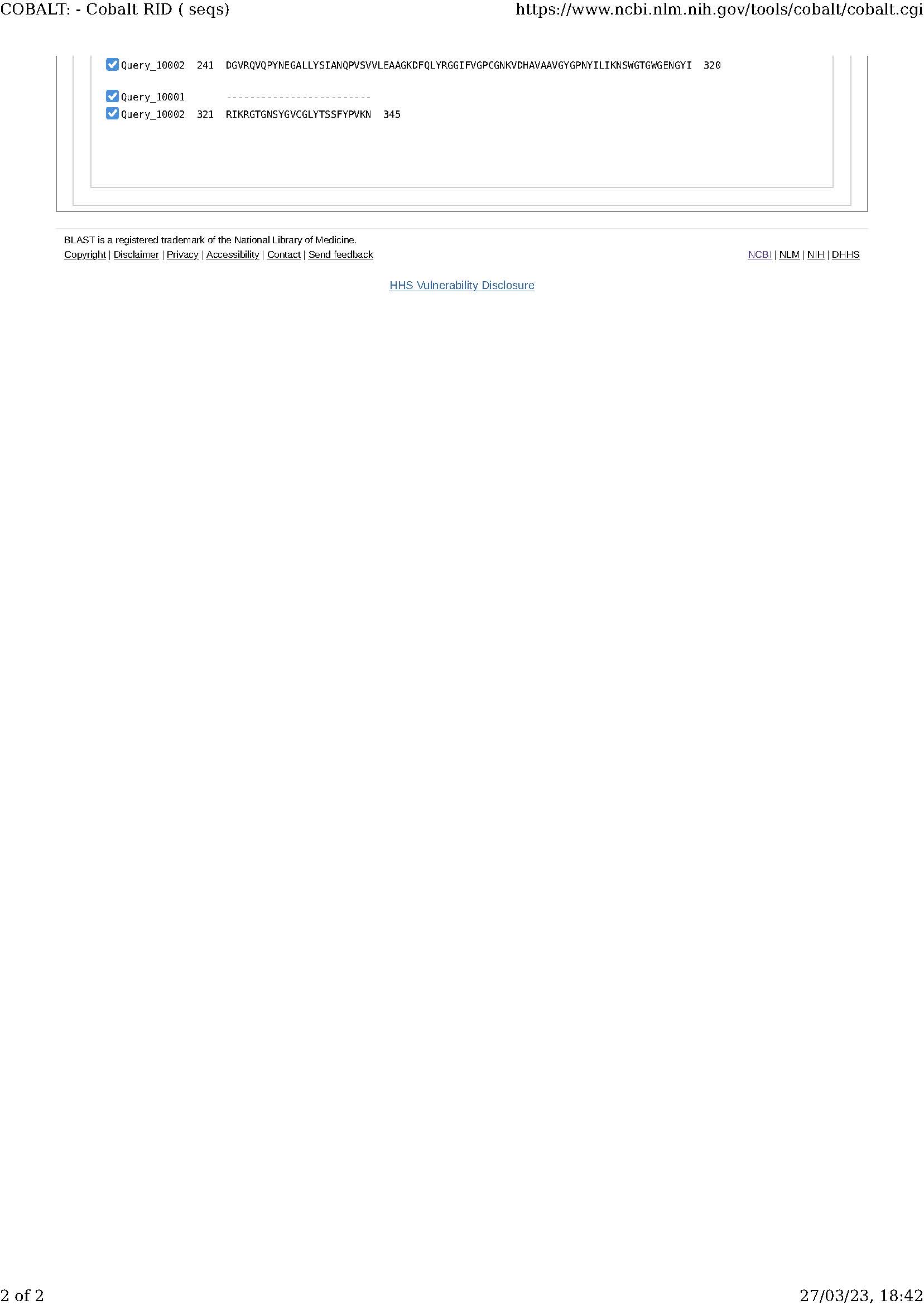


**(C)**


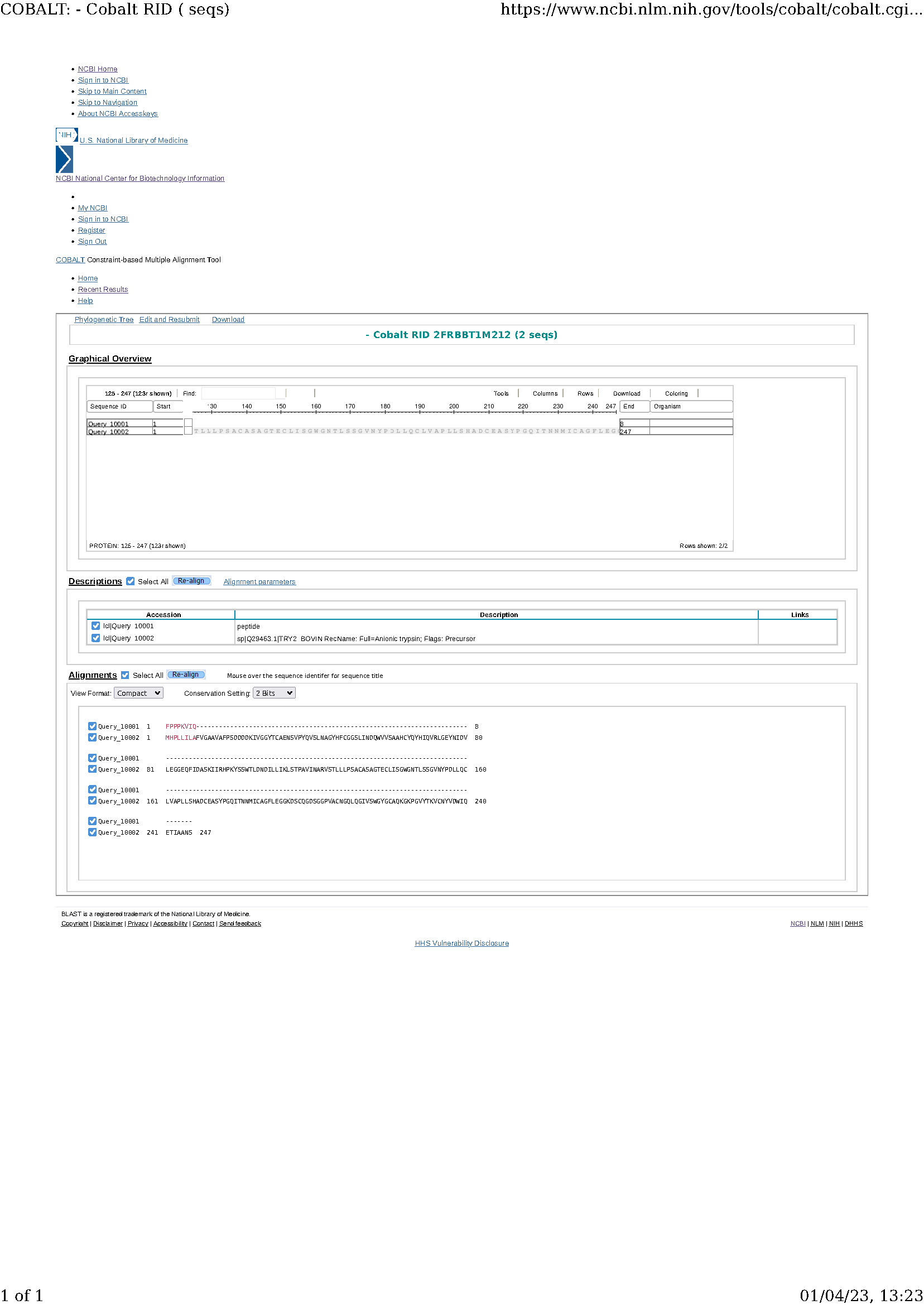


**Figure S2. Protein BLAST (BLASTP) result of the characterized peptide (FPPPKVIQ) with *Vigna aconitifolia***


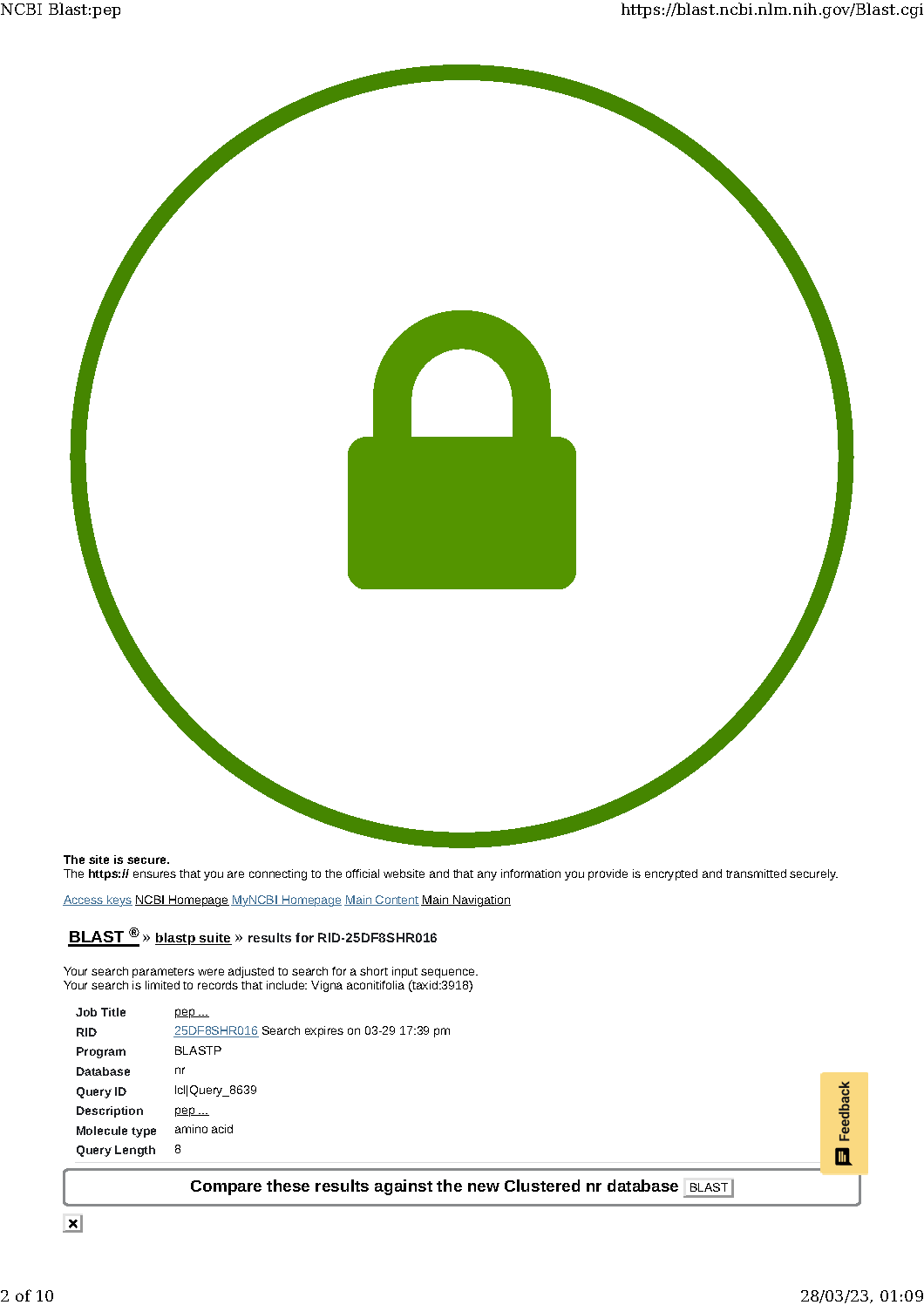


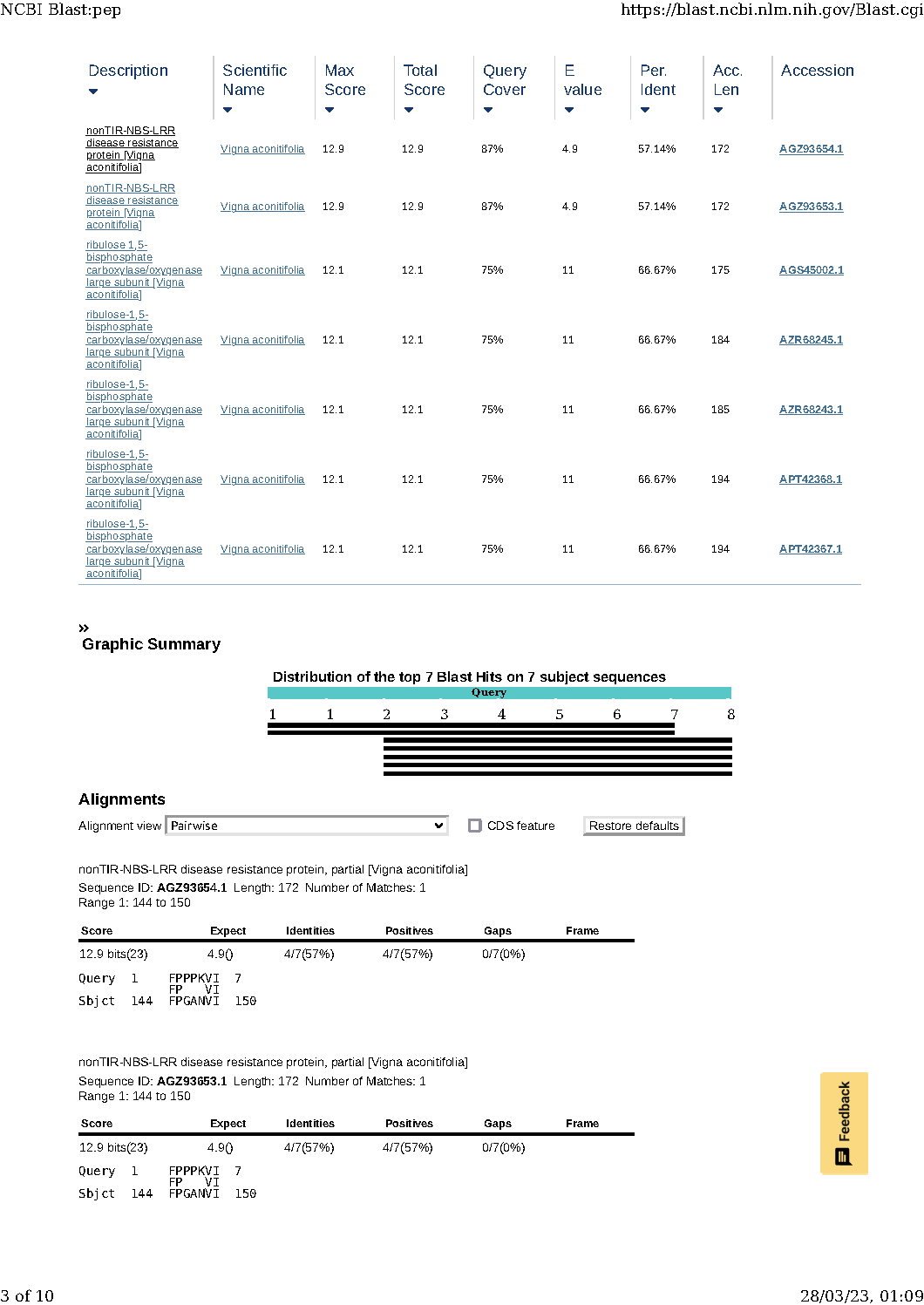


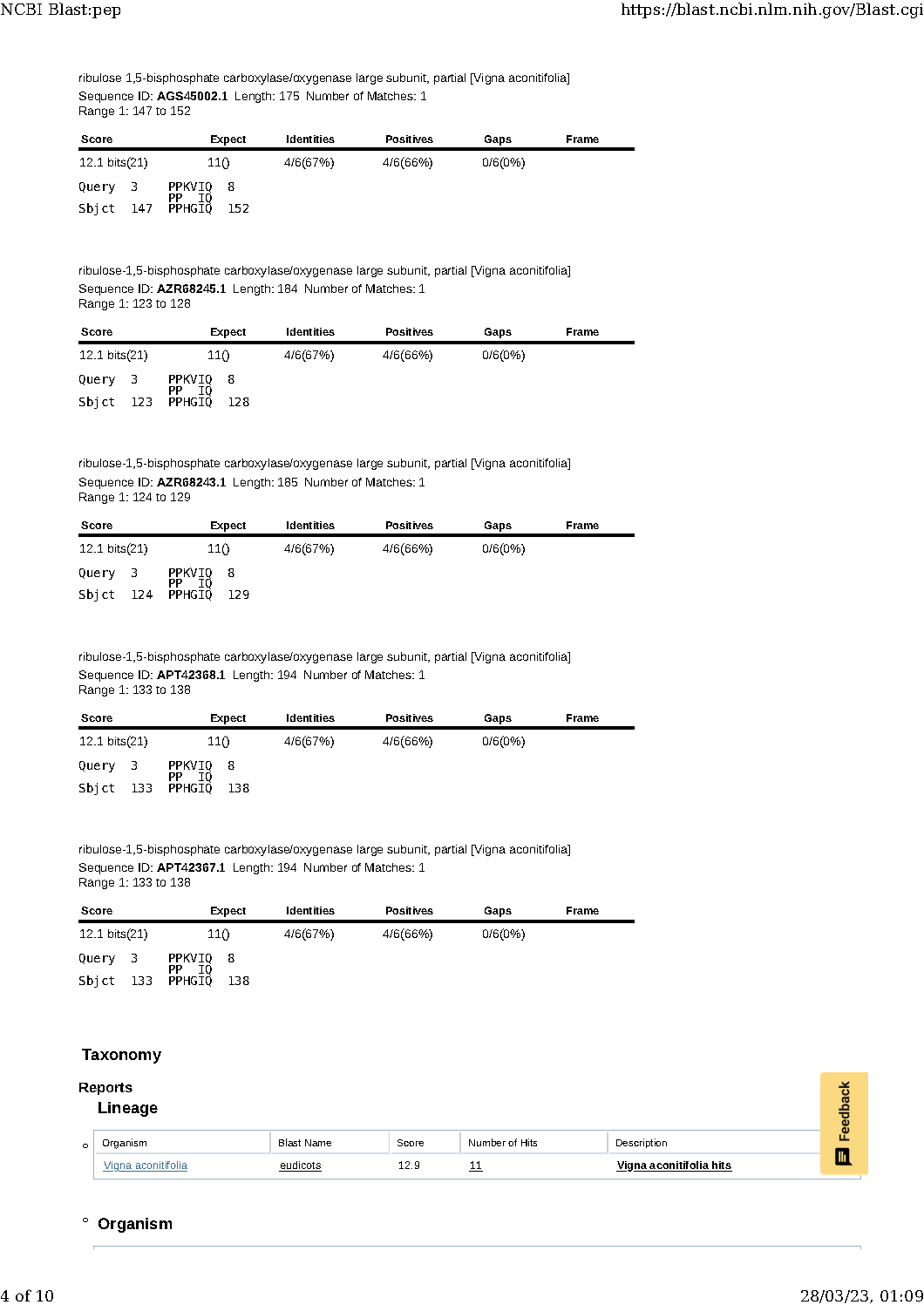


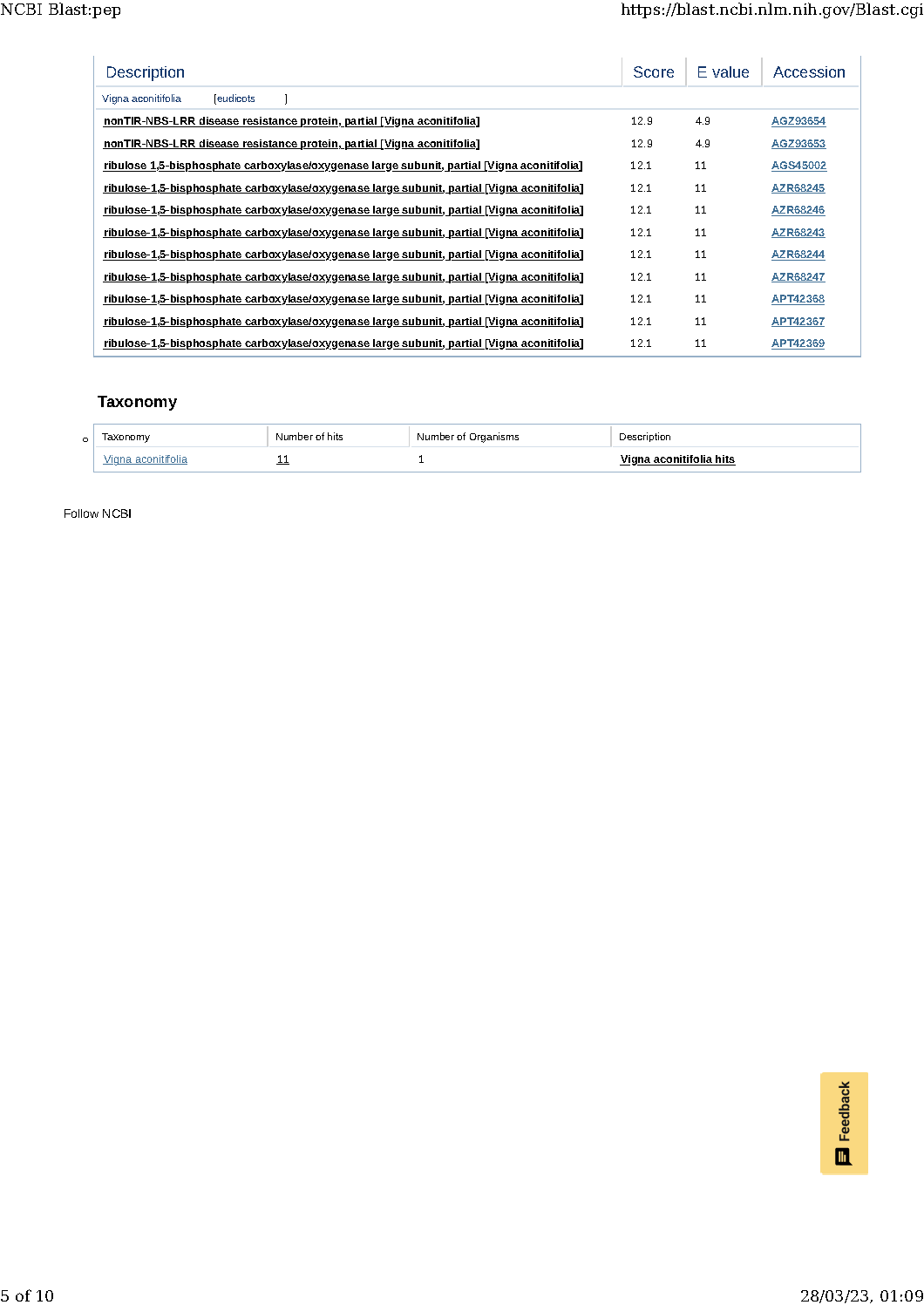

Supplement: Supplementary file 1 [file Data_Sheet_1.docx]
