# Supplementary material for: Release of an encrypted, highly potent ACE-inhibitory peptide by enzymatic hydrolysis of moth bean (Vigna aconitifolia) protein
Source: Front Nutr. 2023 Jun 9;10:1167259. doi: 10.3389/fnut.2023.1167259 (PMC10288869; doi:10.3389/fnut.2023.1167259)
Supplement: Supplementary file 2 [file Data_Sheet_2.pdf]

Supplementary Material 2

Release of an encrypted, highly potent ACE inhibitory peptide by enzymatic hydrolysis of moth bean protein

Nancy Goyal, Sachin N. Hajare[*] and S. Gautam

*** Correspondence:** Corresponding Author: shajare@barc.gov.in

Figure S3. Protein BLAST (BLASTP) result of the characterized peptide (FPPPKVIQ) with other *Vigna* species.

An official website of the United States government

Here's how you know

The .gov means it’s official.
Federal government websites often end in .gov or .mil. Before sharing sensitive information, make sure you’re on a federal government site.

Feedback

The site is secure.
The **https://** ensures that you are connecting to the official website and that any information you provide is encrypted and transmitted securely.

Access keys NCBI Homepage MyNCBI Homepage Main Content Main Navigation

BLAST ® » blastp suite » results for RID-25KXBRE7016

Your search parameters were adjusted to search for a short input sequence.
Your search is limited to records that include: Vigna (taxid:3913)

**Job Title**	pep ...
**RID**	25KXBRE7016 Search expires on 03-29 19:29 pm
**Program**	BLASTP
**Database**	nr
**Query ID**	lcl|Query_53060
**Description**	pep ...
**Molecule type**	amino acid
**Query Length**	8

Feedback

Compare these results against the new Clustered nr database BLAST

×

Descriptions

Description	Scientific Name	Max Score	Total Score	Query Cover	E value	Per. Ident	Acc. Len	Accession
linoleate 9S-lipoxygenase-like [Vigna unguiculata]	Vigna unguiculata	29.9	29.9	100%	0.022	100.00%	867	XP_027907322.1
linoleate 9S-lipoxygenase-like [Vigna radiata]	Vigna radiata	29.9	29.9	100%	0.022	100.00%	867	NP_001304246.1
linoleate 9S-lipoxygenase-like [Vigna umbellata]	Vigna umbellata	29.9	29.9	100%	0.022	100.00%	867	XP_047152047.1
linoleate 9S-lipoxygenase [Vigna angularis]	Vigna angularis	29.9	29.9	100%	0.022	100.00%	867	XP_017411028.1
seed linoleate 9S-lipoxygenase-3 [Vigna unguiculata]	Vigna unguiculata	29.9	29.9	100%	0.022	100.00%	860	XP_027929141.1
seed linoleate 9S-lipoxygenase-3 [Vigna radiata var. radiata]	Vigna radiata var. radiata	29.9	29.9	100%	0.022	100.00%	860	XP_014499690.1
seed linoleate 9S-lipoxygenase-3 [Vigna angularis]	Vigna angularis	29.9	29.9	100%	0.022	100.00%	860	XP_017425282.1
Linoleate 9S-lipoxygenase [Vigna angularis]	Vigna angularis	29.9	29.9	100%	0.022	100.00%	793	KAG2379958.1
Seed linoleate 9S-lipoxygenase-3 [Vigna angularis]	Vigna angularis	29.9	29.9	100%	0.022	100.00%	728	KAG2371189.1
seed linoleate 9S-lipoxygenase-3 [Vigna umbellata]	Vigna umbellata	29.9	29.9	100%	0.022	100.00%	687	XP_047164753.1
lipoxygenase [Vigna unguiculata]	Vigna unguiculata	29.9	29.9	100%	0.022	100.00%	502	QCE02221.1
seed linoleate 9S-lipoxygenase-3-like [Vigna radiata var. radiata]	Vigna radiata var. radiata	29.9	29.9	100%	0.023	100.00%	308	XP_022642514.1
linoleate 9S-lipoxygenase-like [Vigna radiata var. radiata]	Vigna radiata var. radiata	26.9	26.9	100%	0.26	87.50%	858	XP_014501039.1
Cactin [Vigna unguiculata]	Vigna unguiculata	23.5	23.5	87%	4.2	85.71%	707	QCE05878.1
cactin-like [Vigna unguiculata]	Vigna unguiculata	23.5	23.5	87%	4.2	85.71%	662	XP_027936651.1
cactin [Vigna radiata var. radiata]	Vigna radiata var. radiata	23.5	23.5	87%	4.2	85.71%	662	XP_014513749.1
cactin [Vigna umbellata]	Vigna umbellata	23.5	23.5	87%	4.2	85.71%	662	XP_047174620.1
hypothetical protein LR48_Vigan02g076200 [Vigna angularis]	Vigna angularis	23.5	23.5	87%	4.2	85.71%	629	KOM34612.1
linoleate 9S-lipoxygenase-like [Vigna unguiculata]	Vigna unguiculata	23.1	23.1	100%	6.0	75.00%	858	XP_027927434.1
lipoxygenase [Vigna unguiculata]	Vigna unguiculata	23.1	23.1	100%	6.0	75.00%	858	QCE09081.1
linoleate 9S-lipoxygenase [Vigna angularis]	Vigna angularis	23.1	23.1	100%	6.0	75.00%	857	XP_017422959.1
linoleate 9S-lipoxygenase-like [Vigna radiata var. radiata]	Vigna radiata var. radiata	23.1	23.1	100%	6.0	75.00%	857	XP_014501040.1
lipoxygenase [Vigna unguiculata]	Vigna unguiculata	23.1	23.1	100%	6.0	75.00%	856	QCE09083.1
linoleate 9S-lipoxygenase [Vigna radiata var. radiata]	Vigna radiata var. radiata	23.1	23.1	100%	6.0	75.00%	856	XP_014501157.1
linoleate 9S-lipoxygenase [Vigna unguiculata]	Vigna unguiculata	23.1	23.1	100%	6.0	75.00%	856	XP_027929358.1
linoleate 9S-lipoxygenase-like [Vigna angularis]	Vigna angularis	23.1	23.1	100%	6.0	75.00%	856	XP_0174255[illegible]
linoleate 9S-lipoxygenase [Vigna angularis]	Vigna angularis	23.1	23.1	100%	6.0	75.00%	855	XP_0174252[illegible]
linoleate 9S-lipoxygenase [Vigna umbellata]	Vigna umbellata	23.1	23.1	100%	6.0	75.00%	737	XP_047148505.1
E3 ubiquitin-protein ligase RGLG3-like [Vigna unguiculata]	Vigna unguiculata	22.3	22.3	87%	12	87.50%	452	XP_027920351.1

Feedback

Description	Scientific Name	Max Score	Total Score	Query Cover	E value	Per. Ident	Acc. Len	Accession
E3 ubiquitin-protein ligase mind-bomb [Vigna unguiculata]	Vigna unguiculata	22.3	22.3	87%	12	87.50%	452	QCD84854.1
E3 ubiquitin-protein ligase RGLG3 isoform X1 [Vigna radiata var. radiata]	Vigna radiata var. radiata	22.3	22.3	87%	12	87.50%	442	XP_014523792.1
E3 ubiquitin-protein ligase RGLG3 isoform X2 [Vigna radiata var. radiata]	Vigna radiata var. radiata	22.3	22.3	87%	12	87.50%	439	XP_022632568.1
protein PLASTID MOVEMENT IMPAIRED 1-RELATED 1 [Vigna radiata var. radiata]	Vigna radiata var. radiata	21.8	21.8	87%	17	85.71%	1135	XP_014506711.1
protein PLASTID MOVEMENT IMPAIRED 1-RELATED 1-like [Vigna umbellata]	Vigna umbellata	21.8	21.8	87%	17	85.71%	1134	XP_047151724.1
LOW QUALITY PROTEIN: protein PLASTID MOVEMENT IMPAIRED 1-RELATED 1 [Vigna angularis]	Vigna angularis	21.8	21.8	87%	17	85.71%	1133	XP_017428355.1
protein PLASTID MOVEMENT IMPAIRED 1-RELATED 1-like [Vigna unguiculata]	Vigna unguiculata	21.8	21.8	87%	17	85.71%	1124	XP_027918329.1
hypothetical protein LR48_Vigan07g140900 [Vigna angularis]	Vigna angularis	21.8	21.8	87%	17	85.71%	1108	KOM47705.1
Protein PLASTID MOVEMENT IMPAIRED 1-RELATED 1 [Vigna angularis]	Vigna angularis	21.8	21.8	87%	17	85.71%	1092	KAG2407482.1
hypothetical protein VIGAN_01491400 [Vigna angularis var. angularis]	Vigna angularis var. angularis	21.8	21.8	87%	17	85.71%	952	BAT76853.1
seed linoleate 9S-lipoxygenase isoform X1 [Vigna angularis]	Vigna angularis	21.8	21.8	87%	17	85.71%	879	XP_017411037.1
seed linoleate 9S-lipoxygenase isoform X2 [Vigna angularis]	Vigna angularis	21.8	21.8	87%	17	85.71%	877	XP_017411038.1
hypothetical protein LR48_Vigan878s001300 [Vigna angularis]	Vigna angularis	21.8	21.8	87%	17	85.71%	872	KOM30095.1
seed linoleate 9S-lipoxygenase-like [Vigna umbellata]	Vigna umbellata	21.8	21.8	87%	17	85.71%	868	XP_047152050.1
Seed linoleate 9S-lipoxygenase [Vigna angularis]	Vigna angularis	21.8	21.8	87%	17	85.71%	753	KAG2379962.1
serine/arginine repetitive matrix protein 1-like [Vigna unguiculata]	Vigna unguiculata	21.8	21.8	87%	17	85.71%	587	XP_027906026.1
serine/arginine repetitive matrix protein 1-like [Vigna umbellata]	Vigna umbellata	21.8	36.9	87%	17	85.71%	583	XP_047152059.1
uncharacterized protein LOC106773586 [Vigna radiata var. radiata]	Vigna radiata var. radiata	21.8	21.8	87%	17	85.71%	583	XP_014515775.1
uncharacterized protein LOC108320209 isoform X1 [Vigna angularis]	Vigna angularis	21.8	36.9	87%	17	85.71%	580	XP_017407063.1
hypothetical protein LR48_Vigan641s003200 [Vigna angularis]	Vigna angularis	21.4	21.4	87%	25	71.43%	676	KOM29244.1
cactin isoform X1 [Vigna angularis]	Vigna angularis	21.4	21.4	87%	25	71.43%	639	XP_0174100[illegible]
cactin isoform X1 [Vigna radiata var. radiata]	Vigna radiata var. radiata	21.4	21.4	87%	25	71.43%	638	XP_0144962[illegible]
cactin-like isoform X1 [Vigna unguiculata]	Vigna unguiculata	21.4	21.4	87%	25	71.43%	637	XP_0279380[illegible]
cactin isoform X2 [Vigna angularis]	Vigna angularis	21.4	21.4	87%	25	71.43%	539	XP_052728707.1
cactin-like isoform X2 [Vigna unguiculata]	Vigna unguiculata	21.4	21.4	87%	25	71.43%	538	XP_027938055.1

Feedback

Description	Scientific Name	Max Score	Total Score	Query Cover	E value	Per. Ident	Acc. Len	Accession
cactin isoform X2 [Vigna radiata var. radiata]	Vigna radiata var. radiata	21.4	21.4	87%	25	71.43%	538	XP_014496202.1
lipoxygenase [Vigna unguiculata]	Vigna unguiculata	21.0	56.2	100%	35	85.71%	2432	QCE02220.1
lipoxygenase [Vigna unguiculata]	Vigna unguiculata	21.0	42.0	87%	35	85.71%	2409	QCE02434.1
uncharacterized protein LOC106773082 [Vigna radiata var. radiata]	Vigna radiata var. radiata	21.0	38.2	87%	35	85.71%	1742	XP_022642001.1
uncharacterized protein LOC114165656 [Vigna unguiculata]	Vigna unguiculata	21.0	38.2	87%	35	85.71%	1740	XP_027906030.1
uncharacterized protein LOC106773087 [Vigna radiata var. radiata]	Vigna radiata var. radiata	21.0	42.0	87%	35	85.71%	1737	XP_022642004.1
Xaa-Pro aminopeptidase [Vigna unguiculata]	Vigna unguiculata	21.0	21.0	75%	35	83.33%	1022	QCD77747.1
seed linoleate 9S-lipoxygenase-like [Vigna umbellata]	Vigna umbellata	21.0	21.0	87%	35	85.71%	871	XP_047152045.1
seed linoleate 9S-lipoxygenase [Vigna angularis]	Vigna angularis	21.0	21.0	87%	35	85.71%	867	XP_017411029.1
linoleate 9S-lipoxygenase 1-like [Vigna umbellata]	Vigna umbellata	21.0	21.0	87%	35	85.71%	865	XP_047152056.1
linoleate 9S-lipoxygenase 1 [Vigna angularis]	Vigna angularis	21.0	21.0	87%	35	85.71%	865	XP_017407061.1
linoleate 9S-lipoxygenase 1-like [Vigna unguiculata]	Vigna unguiculata	21.0	21.0	87%	35	85.71%	864	XP_027906855.1
linoleate 9S-lipoxygenase 1 [Vigna umbellata]	Vigna umbellata	21.0	21.0	87%	35	85.71%	862	XP_047152057.1
linoleate 9S-lipoxygenase 1 [Vigna unguiculata]	Vigna unguiculata	21.0	21.0	87%	35	85.71%	862	XP_027906856.1
linoleate 9S-lipoxygenase 1 [Vigna angularis]	Vigna angularis	21.0	21.0	87%	35	85.71%	862	XP_017407033.1
Linoleate 9S-lipoxygenase [Vigna angularis]	Vigna angularis	21.0	21.0	87%	35	85.71%	803	KAG2379972.1
Seed linoleate 9S-lipoxygenase [Vigna angularis]	Vigna angularis	21.0	21.0	87%	35	85.71%	694	KAG2379960.1
hypothetical protein VIGAN_09200600 [Vigna angularis var. angularis]	Vigna angularis var. angularis	21.0	21.0	87%	35	85.71%	373	BAT98359.1
microtubule-binding protein TANGLED [Vigna angularis]	Vigna angularis	21.0	21.0	75%	35	83.33%	359	XP_017435853.2
linoleate 9S-lipoxygenase 1-like [Vigna umbellata]	Vigna umbellata	21.0	21.0	87%	35	85.71%	310	XP_047149962.1
microtubule-binding protein TANGLED [Vigna umbellata]	Vigna umbellata	21.0	21.0	75%	35	83.33%	301	XP_047179944.1
microtubule-binding protein TANGLED-like [Vigna unguiculata]	Vigna unguiculata	21.0	21.0	75%	35	83.33%	288	XP_027933789.1
hypothetical protein VIGAN_05151800 [Vigna angularis var. angularis]	Vigna angularis var. angularis	21.0	21.0	75%	35	83.33%	285	BAT88078.1
hypothetical protein DEO72_LG4g1057 [Vigna unguiculata]	Vigna unguiculata	21.0	35.2	100%	35	83.33%	249	QCD90103.1
hypothetical protein LR48_Vigan01g076800 [Vigna angularis]	Vigna angularis	20.6	20.6	87%	50	71.43%	693	KOM31213.1
uncharacterized protein LOC124821985 [Vigna umbellata]	Vigna umbellata	20.6	20.6	75%	50	83.33%	338	XP_0471498[illegible]
receptor-like protein kinase ANXUR2 [Vigna umbellata]	Vigna umbellata	20.6	20.6	75%	50	83.33%	338	XP_0471512[illegible]
receptor-like protein kinase ANXUR2 [Vigna angularis]	Vigna angularis	20.6	20.6	75%	50	83.33%	338	XP_017418678.1

Feedback

Description	Scientific Name	Max Score	Total Score	Query Cover	E value	Per. Ident	Acc. Len	Accession
receptor-like serine/threonine-protein kinase ALE2 isoform X1 [Vigna unguiculata]	Vigna unguiculata	20.2	20.2	62%	71	100.00%	1279	XP_027902850.1
receptor-like serine/threonine-protein kinase ALE2 isoform X2 [Vigna unguiculata]	Vigna unguiculata	20.2	20.2	62%	71	100.00%	1278	XP_027902851.1
receptor-like serine/threonine-protein kinase ALE2 isoform X3 [Vigna unguiculata]	Vigna unguiculata	20.2	20.2	62%	71	100.00%	1239	XP_027902852.1
interleukin-1 receptor-associated kinase 4 [Vigna unguiculata]	Vigna unguiculata	20.2	20.2	62%	71	100.00%	1214	QCE13516.1
FT-interacting protein 3 [Vigna angularis]	Vigna angularis	20.2	20.2	75%	71	83.33%	1014	XP_017407705.1
FT-interacting protein 7-like [Vigna umbellata]	Vigna umbellata	20.2	20.2	75%	71	83.33%	1014	XP_047160656.1
FT-interacting protein 1 [Vigna radiata var. radiata]	Vigna radiata var. radiata	20.2	20.2	75%	71	83.33%	1014	XP_014496606.1
FT-interacting protein 3-like [Vigna unguiculata]	Vigna unguiculata	20.2	20.2	75%	71	83.33%	1012	XP_027928010.1
phosphatidylserine decarboxylase [Vigna unguiculata]	Vigna unguiculata	20.2	20.2	75%	71	83.33%	1012	QCE12291.1
hypothetical protein VIGAN_04382100 [Vigna angularis var. angularis]	Vigna angularis var. angularis	20.2	20.2	62%	71	100.00%	901	BAT86194.1
protein transport protein SEC23 [Vigna umbellata]	Vigna umbellata	20.2	20.2	62%	71	100.00%	873	XP_047172003.1
protein transport protein SEC23 [Vigna angularis]	Vigna angularis	20.2	20.2	62%	71	100.00%	873	XP_017418498.1
uncharacterized protein HKW66_Vig0057290 [Vigna angularis]	Vigna angularis	20.2	20.2	62%	71	100.00%	870	KAG2406473.1
hypothetical protein LR48_Vigan03g272600 [Vigna angularis]	Vigna angularis	20.2	20.2	62%	71	100.00%	846	KOM39344.1
hypothetical protein LR48_Vigan09g151400 [Vigna angularis]	Vigna angularis	20.2	67.5	87%	71	85.71%	841	KOM52855.1
uncharacterized protein LOC108343573 [Vigna angularis]	Vigna angularis	20.2	67.5	87%	71	85.71%	818	XP_017437422.1
uncharacterized protein LOC128195279 [Vigna angularis]	Vigna angularis	20.2	20.2	62%	71	100.00%	810	XP_052728479.1
uncharacterized protein LOC124822029 [Vigna umbellata]	Vigna umbellata	20.2	68.3	87%	71	85.71%	808	XP_047149936.1

»

Graphic Summary

Feedback

Distribution of the top 118 Blast Hits on 100 subject sequences

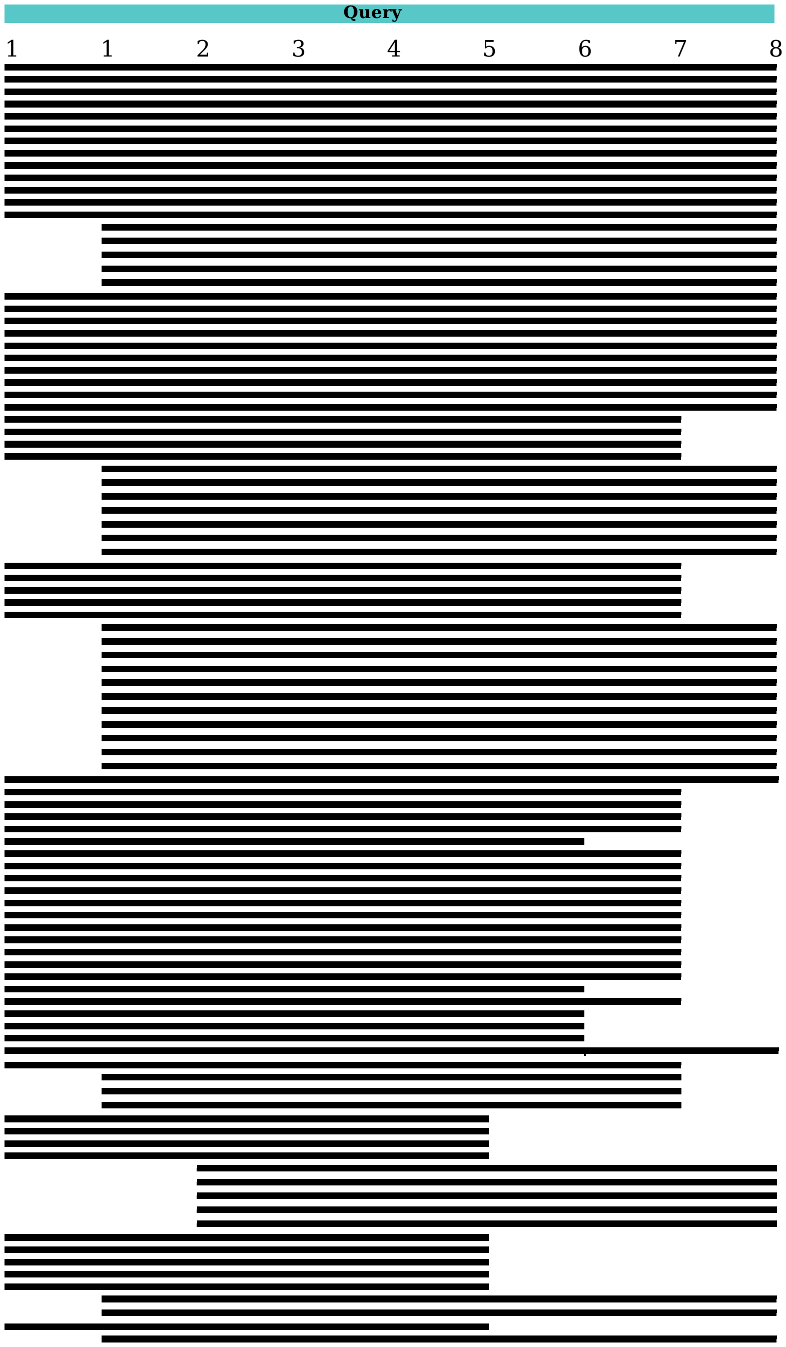

Alignments

Alignment view Pairwise ☐ CDS feature Restore defaults

linoleate 9S-lipoxygenase-like [Vigna unguiculata]

Sequence ID: **XP_027907322.1** Length: 867 Number of Matches: 1

Range 1: 354 to 361

Score	Expect	Identities	Positives	Gaps	Frame
29.9 bits(63)	0.022()	8/8(100%)	8/8(100%)	0/8(0%)	

```
Query  1    FPPPKVIQ  8
            FPPPKVIQ
Sbjct  354  FPPPKVIQ  361
```

Feedback

linoleate 9S-lipoxygenase-like [Vigna radiata]

Sequence ID: **NP_001304246.1** Length: 867 Number of Matches: 1

Range 1: 354 to 361

Score	Expect	Identities	Positives	Gaps	Frame
29.9 bits(63)	0.022()	8/8(100%)	8/8(100%)	0/8(0%)	

```
Query  1    FPPPKVIQ  8
            FPPPKVIQ
Sbjct  354  FPPPKVIQ  361
```

linoleate 9S-lipoxygenase-like [Vigna umbellata]

Sequence ID: **XP_047152047.1** Length: 867 Number of Matches: 1

Range 1: 354 to 361

Score	Expect	Identities	Positives	Gaps	Frame
29.9 bits(63)	0.022()	8/8(100%)	8/8(100%)	0/8(0%)	

```
Query  1    FPPPKVIQ  8
            FPPPKVIQ
Sbjct  354  FPPPKVIQ  361
```

linoleate 9S-lipoxygenase [Vigna angularis]

Sequence ID: **XP_017411028.1** Length: 867 Number of Matches: 1

Range 1: 354 to 361

Score	Expect	Identities	Positives	Gaps	Frame
29.9 bits(63)	0.022()	8/8(100%)	8/8(100%)	0/8(0%)	

```
Query  1    FPPPKVIQ  8
            FPPPKVIQ
Sbjct  354  FPPPKVIQ  361
```

seed linoleate 9S-lipoxygenase-3 [Vigna unguiculata]

Sequence ID: **XP_027929141.1** Length: 860 Number of Matches: 1

Range 1: 348 to 355

Score	Expect	Identities	Positives	Gaps	Frame
29.9 bits(63)	0.022()	8/8(100%)	8/8(100%)	0/8(0%)	

```
Query  1    FPPPKVIQ  8
            FPPPKVIQ
Sbjct  348  FPPPKVIQ  355
```

Taxonomy

Reports

Lineage

Organism	Blast Name	Score	Number of Hits	Description
Vigna	eudicots		152	
.Vigna unguiculata	eudicots	29.9	37	**Vigna unguiculata hits**
.Vigna radiata	eudicots	29.9	2	**Vigna radiata hits**
.Vigna umbellata	eudicots	29.9	20	**Vigna umbellata hits**
.Vigna angularis	eudicots	29.9	59	**Vigna angularis hits**
.Vigna angularis var. angularis	eudicots	29.9	14	**Vigna angularis var. angularis hits**
.Vigna radiata var. radiata	eudicots	29.9	20	**Vigna radiata var. radiata hits**

Organism

Description	Score	E value	Accession
Vigna unguiculata (cowpea) [eudicots]			
**linoleate 9S-lipoxygenase-like [Vigna unguiculata]**	29.9	0.022	XP_027907[illegible]
**seed linoleate 9S-lipoxygenase-3 [Vigna unguiculata]**	29.9	0.022	XP_027929[illegible]
**lipoxygenase [Vigna unguiculata]**	29.9	0.022	QCE09080
**lipoxygenase [Vigna unguiculata]**	29.9	0.022	QCE02221
**Cactin [Vigna unguiculata]**	23.5	4.2	QCE05878
**cactin-like [Vigna unguiculata]**	23.5	4.2	XP_027936651
**linoleate 9S-lipoxygenase-like [Vigna unguiculata]**	23.1	6.0	XP_027927434

Feedback

Description	Score	E value	Accession
lipoxygenase [Vigna unguiculata]	23.1	6.0	QCE09081
lipoxygenase [Vigna unguiculata]	23.1	6.0	QCE09083
linoleate 9S-lipoxygenase [Vigna unguiculata]	23.1	6.0	XP_027929358
E3 ubiquitin-protein ligase RGLG3-like [Vigna unguiculata]	22.3	12	XP_027920351
E3 ubiquitin-protein ligase RGLG3-like [Vigna unguiculata]	22.3	12	XP_027920352
E3 ubiquitin-protein ligase RGLG3-like [Vigna unguiculata]	22.3	12	XP_027920354
E3 ubiquitin-protein ligase RGLG3-like [Vigna unguiculata]	22.3	12	XP_027920355
E3 ubiquitin-protein ligase RGLG3-like [Vigna unguiculata]	22.3	12	XP_027920356
E3 ubiquitin-protein ligase mind-bomb [Vigna unguiculata]	22.3	12	QCD84854
protein PLASTID MOVEMENT IMPAIRED 1-RELATED 1-like [Vigna unguiculata]	21.8	17	XP_027918329
hypothetical protein DEO72_LG2g577 [Vigna unguiculata]	21.8	17	QCD80256
serine/arginine repetitive matrix protein 1-like [Vigna unguiculata]	21.8	17	XP_027906026
cactin-like isoform X1 [Vigna unguiculata]	21.4	25	XP_027938045
Cactin [Vigna unguiculata]	21.4	25	QCD97991
cactin-like isoform X2 [Vigna unguiculata]	21.4	25	XP_027938055
lipoxygenase [Vigna unguiculata]	21.0	35	QCE02220
lipoxygenase [Vigna unguiculata]	21.0	35	QCE02434
uncharacterized protein LOC114165656 [Vigna unguiculata]	21.0	35	XP_027906030
Xaa-Pro aminopeptidase [Vigna unguiculata]	21.0	35	QCD77747
linoleate 9S-lipoxygenase 1-like [Vigna unguiculata]	21.0	35	XP_027906855
linoleate 9S-lipoxygenase 1 [Vigna unguiculata]	21.0	35	XP_027906856
microtubule-binding protein TANGLED-like [Vigna unguiculata]	21.0	35	XP_027933789
hypothetical protein DEO72_LG4g1057 [Vigna unguiculata]	21.0	35	QCD90103
receptor-like serine/threonine-protein kinase ALE2 isoform X1 [Vigna unguiculata]	20.2	71	XP_027902850
receptor-like serine/threonine-protein kinase ALE2 isoform X2 [Vigna unguiculata]	20.2	71	XP_027902851
receptor-like serine/threonine-protein kinase ALE2 isoform X3 [Vigna unguiculata]	20.2	71	XP_027902852
interleukin-1 receptor-associated kinase 4 [Vigna unguiculata]	20.2	71	QCE13516
FT-interacting protein 3-like [Vigna unguiculata]	20.2	71	XP_027928010
FT-interacting protein 3-like [Vigna unguiculata]	20.2	71	XP_027928011
phosphatidylserine decarboxylase [Vigna unguiculata]	20.2	71	QCE12291
Vigna radiata [eudicots]			
linoleate 9S-lipoxygenase-like [Vigna radiata]	29.9	0.022	NP_001304246
lipoxygenase [Vigna radiata]	29.9	0.022	AGS94394
Vigna umbellata [eudicots]			
linoleate 9S-lipoxygenase-like [Vigna umbellata]	29.9	0.022	XP_047152047
seed linoleate 9S-lipoxygenase-3, partial [Vigna umbellata]	29.9	0.022	XP_047164753
cactin [Vigna umbellata]	23.5	4.2	XP_047174620
linoleate 9S-lipoxygenase [Vigna umbellata]	23.1	6.0	XP_047148505
protein PLASTID MOVEMENT IMPAIRED 1-RELATED 1-like [Vigna umbellata]	21.8	17	XP_047151724
seed linoleate 9S-lipoxygenase-like [Vigna umbellata]	21.8	17	XP_047152050
serine/arginine repetitive matrix protein 1-like [Vigna umbellata]	21.8	17	XP_047152059
seed linoleate 9S-lipoxygenase-like [Vigna umbellata]	21.0	35	XP_047152045
linoleate 9S-lipoxygenase 1-like [Vigna umbellata]	21.0	35	XP_047152056
linoleate 9S-lipoxygenase 1 [Vigna umbellata]	21.0	35	XP_047152057
linoleate 9S-lipoxygenase 1-like, partial [Vigna umbellata]	21.0	35	XP_047149962
microtubule-binding protein TANGLED, partial [Vigna umbellata]	21.0	35	XP_047179944
uncharacterized protein LOC124821985 [Vigna umbellata]	20.6	50	XP_047149[illegible]
receptor-like protein kinase ANXUR2 [Vigna umbellata]	20.6	50	XP_047151[illegible]
FT-interacting protein 7-like [Vigna umbellata]	20.2	71	XP_047160[illegible]
protein transport protein SEC23 [Vigna umbellata]	20.2	71	XP_047172[illegible]
protein transport protein SEC23 [Vigna umbellata]	20.2	71	XP_047172[illegible]
protein transport protein SEC23 [Vigna umbellata]	20.2	71	XP_047172005
protein transport protein SEC23 [Vigna umbellata]	20.2	71	XP_047172006
uncharacterized protein LOC124822029 [Vigna umbellata]	20.2	71	XP_047149936

Feedback

Description	Score	E value	Accession
Vigna angularis (adzuki bean) [eudicots]			
linoleate 9S-lipoxygenase [Vigna angularis]	29.9	0.022	XP_017411028
hypothetical protein LR48_Vigan878s000900 [Vigna angularis]	29.9	0.022	KOM30091
seed linoleate 9S-lipoxygenase-3 [Vigna angularis]	29.9	0.022	XP_017425282
hypothetical protein LR48_Vigan05g201600 [Vigna angularis]	29.9	0.022	KOM44411
Linoleate 9S-lipoxygenase [Vigna angularis]	29.9	0.022	KAG2379958
Seed linoleate 9S-lipoxygenase-3 [Vigna angularis]	29.9	0.022	KAG2371189
hypothetical protein LR48_Vigan02g076200 [Vigna angularis]	23.5	4.2	KOM34612
linoleate 9S-lipoxygenase [Vigna angularis]	23.1	6.0	XP_017422959
Linoleate 9S-lipoxygenase-4 [Vigna angularis]	23.1	6.0	KAG2371192
hypothetical protein LR48_Vigan05g201800 [Vigna angularis]	23.1	6.0	KOM44413
linoleate 9S-lipoxygenase-like [Vigna angularis]	23.1	6.0	XP_017425551
Linoleate 9S-lipoxygenase-4 [Vigna angularis]	23.1	6.0	KAG2371190
linoleate 9S-lipoxygenase [Vigna angularis]	23.1	6.0	XP_017425294
Linoleate 9S-lipoxygenase-4 [Vigna angularis]	23.1	6.0	KAG2371191
hypothetical protein LR48_Vigan05g201700 [Vigna angularis]	23.1	6.0	KOM44412
LOW QUALITY PROTEIN: protein PLASTID MOVEMENT IMPAIRED 1-RELATED 1 [Vigna angularis]	21.8	17	XP_017428355
hypothetical protein LR48_Vigan07g140900 [Vigna angularis]	21.8	17	KOM47705
Protein PLASTID MOVEMENT IMPAIRED 1-RELATED 1 [Vigna angularis]	21.8	17	KAG2407482
seed linoleate 9S-lipoxygenase isoform X1 [Vigna angularis]	21.8	17	XP_017411037
seed linoleate 9S-lipoxygenase isoform X2 [Vigna angularis]	21.8	17	XP_017411038
hypothetical protein LR48_Vigan878s001300 [Vigna angularis]	21.8	17	KOM30095
Seed linoleate 9S-lipoxygenase [Vigna angularis]	21.8	17	KAG2379962
uncharacterized protein LOC108320209 isoform X1 [Vigna angularis]	21.8	17	XP_017407063
uncharacterized protein LOC108320209 isoform X1 [Vigna angularis]	21.8	17	XP_052723941
uncharacterized protein LOC108320209 isoform X1 [Vigna angularis]	21.8	17	XP_052723942
uncharacterized protein LOC108320209 isoform X1 [Vigna angularis]	21.8	17	XP_052723943
uncharacterized protein HKW66_Vig0167530 [Vigna angularis]	21.8	17	KAG2379974
hypothetical protein LR48_Vigan346s001900 [Vigna angularis]	21.8	17	KOM26937
hypothetical protein LR48_Vigan641s003200 [Vigna angularis]	21.4	25	KOM29244
cactin isoform X1 [Vigna angularis]	21.4	25	XP_017410064
Cactin protein [Vigna angularis]	21.4	25	KAG2406124
cactin isoform X2 [Vigna angularis]	21.4	25	XP_052728707
seed linoleate 9S-lipoxygenase [Vigna angularis]	21.0	35	XP_017411029
hypothetical protein LR48_Vigan878s001100 [Vigna angularis]	21.0	35	KOM30093
linoleate 9S-lipoxygenase 1 [Vigna angularis]	21.0	35	XP_017407061
Linoleate 9S-lipoxygenase [Vigna angularis]	21.0	35	KAG2379971
hypothetical protein LR48_Vigan346s002300 [Vigna angularis]	21.0	35	KOM26941
linoleate 9S-lipoxygenase 1 [Vigna angularis]	21.0	35	XP_017407033
hypothetical protein LR48_Vigan346s002200 [Vigna angularis]	21.0	35	KOM26940
Linoleate 9S-lipoxygenase [Vigna angularis]	21.0	35	KAG2379972
Seed linoleate 9S-lipoxygenase [Vigna angularis]	21.0	35	KAG2379960
microtubule-binding protein TANGLED [Vigna angularis]	21.0	35	XP_017435853
hypothetical protein LR48_Vigan09g151900 [Vigna angularis]	21.0	35	KOM52860
hypothetical protein LR48_Vigan01g076800 [Vigna angularis]	20.6	50	KOM31213
receptor-like protein kinase ANXUR2 [Vigna angularis]	20.6	50	XP_017418678
Receptor-like protein [Vigna angularis]	20.6	50	KAG240399[illegible]
hypothetical protein LR48_Vigan03g017400 [Vigna angularis]	20.6	50	KOM36793
FT-interacting protein 3 [Vigna angularis]	20.2	71	XP_017407[illegible]
FT-interacting protein [Vigna angularis]	20.2	71	KAG240434[illegible]
hypothetical protein LR48_Vigan412s000400 [Vigna angularis]	20.2	71	KOM27462
protein transport protein SEC23 [Vigna angularis]	20.2	71	XP_017418498
protein transport protein SEC23 [Vigna angularis]	20.2	71	XP_017418500
protein transport protein SEC23 [Vigna angularis]	20.2	71	XP_017418501

Feedback

Description	Score	E value	Accession
protein transport protein SEC23 [Vigna angularis]	20.2	71	XP_017418502
uncharacterized protein HKW66_Vig0057290 [Vigna angularis]	20.2	71	KAG2406473
hypothetical protein LR48_Vigan03g272600 [Vigna angularis]	20.2	71	KOM39344
hypothetical protein LR48_Vigan09g151400 [Vigna angularis]	20.2	71	KOM52855
uncharacterized protein LOC108343573 [Vigna angularis]	20.2	71	XP_017437422
uncharacterized protein LOC128195279 [Vigna angularis]	20.2	71	XP_052728479
Vigna angularis var. angularis [eudicots]			
hypothetical protein VIGAN_09201000 [Vigna angularis var. angularis]	29.9	0.022	BAT98363
hypothetical protein VIGAN_07035200 [Vigna angularis var. angularis]	29.9	0.022	BAT91730
hypothetical protein VIGAN_07035300 [Vigna angularis var. angularis]	23.1	6.0	BAT91731
hypothetical protein VIGAN_07035400 [Vigna angularis var. angularis]	23.1	6.0	BAT91732
hypothetical protein VIGAN_01491400 [Vigna angularis var. angularis]	21.8	17	BAT76853
hypothetical protein VIGAN_09199400 [Vigna angularis var. angularis]	21.8	17	BAT98347
hypothetical protein VIGAN_04337500 [Vigna angularis var. angularis]	21.4	25	BAT85788
hypothetical protein VIGAN_09199700 [Vigna angularis var. angularis]	21.0	35	BAT98350
hypothetical protein VIGAN_09199600 [Vigna angularis var. angularis]	21.0	35	BAT98349
hypothetical protein VIGAN_09200600, partial [Vigna angularis var. angularis]	21.0	35	BAT98359
hypothetical protein VIGAN_05151800 [Vigna angularis var. angularis]	21.0	35	BAT88078
hypothetical protein VIGAN_04084000 [Vigna angularis var. angularis]	20.2	71	BAT83653
hypothetical protein VIGAN_04382100 [Vigna angularis var. angularis]	20.2	71	BAT86194
hypothetical protein VIGAN_05152400 [Vigna angularis var. angularis]	20.2	71	BAT88084
Vigna radiata var. radiata (mung bean) [eudicots]			
seed linoleate 9S-lipoxygenase-3 [Vigna radiata var. radiata]	29.9	0.022	XP_014499690
seed linoleate 9S-lipoxygenase-3 [Vigna radiata var. radiata]	29.9	0.022	XP_022637181
seed linoleate 9S-lipoxygenase-3-like [Vigna radiata var. radiata]	29.9	0.023	XP_022642514
linoleate 9S-lipoxygenase-like [Vigna radiata var. radiata]	26.9	0.26	XP_014501039
cactin [Vigna radiata var. radiata]	23.5	4.2	XP_014513749
linoleate 9S-lipoxygenase-like [Vigna radiata var. radiata]	23.1	6.0	XP_014501040
linoleate 9S-lipoxygenase [Vigna radiata var. radiata]	23.1	6.0	XP_014501157
E3 ubiquitin-protein ligase RGLG3 isoform X1 [Vigna radiata var. radiata]	22.3	12	XP_014523792
E3 ubiquitin-protein ligase RGLG3 isoform X1 [Vigna radiata var. radiata]	22.3	12	XP_014523793
E3 ubiquitin-protein ligase RGLG3 isoform X2 [Vigna radiata var. radiata]	22.3	12	XP_022632568
protein PLASTID MOVEMENT IMPAIRED 1-RELATED 1 [Vigna radiata var. radiata]	21.8	17	XP_014506711
uncharacterized protein LOC106773586 [Vigna radiata var. radiata]	21.8	17	XP_014515775
uncharacterized protein LOC106773586 [Vigna radiata var. radiata]	21.8	17	XP_022642039
cactin isoform X1 [Vigna radiata var. radiata]	21.4	25	XP_014496201
cactin isoform X2 [Vigna radiata var. radiata]	21.4	25	XP_014496202
uncharacterized protein LOC106773082 [Vigna radiata var. radiata]	21.0	35	XP_022642001
uncharacterized protein LOC106773087 [Vigna radiata var. radiata]	21.0	35	XP_022642004
FT-interacting protein 1 [Vigna radiata var. radiata]	20.2	71	XP_014496606
FT-interacting protein 1 [Vigna radiata var. radiata]	20.2	71	XP_022635565
FT-interacting protein 1 [Vigna radiata var. radiata]	20.2	71	XP_022635566

Taxonomy

Taxonomy	Number of hits	Number of Organisms	Description
Vigna	152	6	
. Vigna unguiculata	37	1	Vigna unguiculata hits
. Vigna radiata	2	2	Vigna radiata hits
.. Vigna radiata var. radiata	20	1	Vigna radiata var. radiata hits
. Vigna umbellata	20	1	Vigna umbellata hits
. Vigna angularis	59	2	Vigna angularis hits
.. Vigna angularis var. angularis	14	1	Vigna angularis var. angularis hits

Feedback

Top
Follow NCBI

Feedback

Feedback

Feedback

Feedback

Connect with NLM

National Library of Medicine
8600 Rockville Pike
Bethesda, MD 20894

Web Policies
FOIA
HHS Vulnerability Disclosure

Help
Accessibility
Careers

- NLM
- NIH
- HHS
- USA.gov

Feedback
